# Structural brain improvements following individually tailored serious exergame-based training in mild neurocognitive disorders: exploratory randomized controlled trial

**DOI:** 10.1186/s13195-025-01835-2

**Published:** 2025-09-08

**Authors:** Patrick Manser, Michael Rosio, André Schmidt, Lars Michels, Eling D. de Bruin

**Affiliations:** 1 Motor Control and Learning Group, Institute of Human Movement Sciences and Sport, Department of Health Sciences and Technology, ETH Zurich, Leopold-Ruzicka-Weg 4, Zurich, 8093 Switzerland; 2 Department of Neurobiology, Care Sciences, and Society, Division of Physiotherapy, Karolinska Institutet, Alfred Nobels Allé 23, Huddinge, 14183 Sweden; 3 Clinical Neuroscience Center, Department of Neuroradiology, University Hospital Zurich, Sternwartstrasse 6, Zurich, 8006 Switzerland; 4 Department of Clinical Research, University of Basel, Spitalstrasse 8/12, Basel, 4031 Switzerland; 5 Neuroscience Center Zurich, University of Zurich and ETH Zurich, Winterthurerstrasse 190, Zurich, 8057 Switzerland; 6 Department of Health, OST - Eastern Swiss University of Applied Sciences, Rosenbergstrasse 59, St. Gallen, 9001 Switzerland

**Keywords:** Cognition, Cognitive impairment, Digital health, Effectiveness, Exercise, Hippocampus, Magnetic resonance imaging, Neuroplasticity, Neuroscience, Technology

## Abstract

**Introduction:**

Exergame-based training is emerging as the most effective exercise modality for improving cognition, yet its neural correlates remain largely unexplored. This study explored gray matter (GM) and white matter (WM) changes following the addition of ‘Brain-IT’ training to usual care in mild neurocognitive disorder (mNCD) and their associations with cognitive performance changes.

**Methods:**

We included 41 participants with mNCD, randomized to either the intervention (‘Brain-IT’ training + usual care) or the control (usual care only) group. ‘Brain-IT’ is a holistic, individually tailored *“exercise as medicine”* program for secondary mNCD prevention delivered through serious exergames. T1-weighted and diffusion tensor imaging data were analyzed via standard neuroimaging analysis pipelines (FreeSurfer, tract-based spatial statistics) to assess GM/WM volumes in predefined regions of interest and WM integrity at the voxel-to-voxel level. Intervention-related changes were explored via analyses of covariance, focusing on effect size estimates. One-sided bivariate correlation analyses explored associations between changes in brain structure and cognitive performance.

**Results:**

Complete datasets from 30 study participants (72.0 ± 8.6 years; 27% females) were available. 87% of participants had biomarker-supported characterization of mNCD etiology– mostly Alzheimer’s (62%). Significant moderate to large effects (partial eta-squared = 0.109 to 0.187) on GM/WM volumes were observed in the right and total hippocampus, thalamus, and anterior cingulate cortex in favor of ‘Brain-IT’ training. Hippocampal and thalamic changes correlated with improvements in verbal delayed recall. Protective effects on WM integrity, which correlated with cognitive improvements, were also observed, mainly around the thalamic radiation and the corpus callosum.

**Conclusion:**

This is the first RCT showing that a co-designed, purpose-developed, and individually tailored exergame-based training may positively impact brain structures affected in mNCD, with potential associations suggestive of a causal link to cognitive improvements. Since hippocampal atrophy is a hallmark of Alzheimer’s disease with high prognostic value for disease progression, our observations may be a first indication of a potential disease-modifying role of ‘Brain-IT’ training. However, adequately powered and hypothesis-driven studies are needed to build on these initial exploratory findings and better understand the neurobiological effects of exergame-based training.

**Trial registration:**

ClinicalTrials.gov (NCT05387057; date of registration: May 18, 2022): https://clinicaltrials.gov/ct2/show/NCT05387057.

**Supplementary Information:**

The online version contains supplementary material available at 10.1186/s13195-025-01835-2.

## Background

The brain structure of individuals with mild neurocognitive disorder (mNCD), also referred to as mild cognitive impairment [1], is typically characterized by accelerated brain atrophy and disruptions in white matter (WM) microstructure, which exceed those observed in normal aging [2–4]. These structural abnormalities are proportional to the disease stage [3–6], may vary depending on the etiology of mNCD [2], and can be detected mainly in the medial temporal (e.g., hippocampus) and frontal lobes (e.g., anterior cingulate cortex (ACC) and prefrontal cortex (PFC)), as well as their interconnections (e.g., corpus callosum (CC) and fornix) [2–4, 6, 7]. Hippocampal atrophy is a hallmark of Alzheimer’s disease that ranges on a continuum from healthy aging to mNCD and dementia, similar to other biomarkers of disease pathology (e.g., cerebrospinal fluid biomarkers such as the Aβ42 level, tau, or p-tau) or cognitive performance, with high prognostic value for disease progression [3–6]. Consequently, effective interventions for the secondary prevention of mNCD should have a protective effect on these brain structures that are vulnerable to neurodegenerative processes in mNCD.

Multicomponent physical and motor-cognitive training may represent the predominant nonpharmacological intervention that effectively mitigates cognitive decline in mNCD [8]. Physical training has been proposed to operate through various disease-modifying mechanisms in mNCD. These include multisystem effects that reduce mNCD-related neuropathological damage and promote synaptogenesis, neurogenesis, and angiogenesis [9, 10]. The activation of neurogenesis plays a pivotal role in decelerating, restoring, and rejuvenating structural pathways associated with hippocampal aging, mitigating age- or disease-related abnormalities, and contributing to improved hippocampus-dependent cognition [11, 12]. The simultaneous execution of physical and cognitive exercises supports and stabilizes these neuroplastic processes, facilitating the survival and integration of new neuronal structures in brain circuits and leading to positive synergistic effects [13, 14]. Additionally, it can help maintain or increase brain and cognitive reserve, empowering individuals to retain independence in everyday functioning despite neurodegenerative changes [10, 15]. As a result, physical and/or motor-cognitive training is recommended for the secondary prevention of mNCD [16], with a global consensus on its relevance for enhancing longevity and dementia prevention [8].

Innovations in interactive and gamified digital health technologies, such as exergames, offer additional benefits for implementing structured physical and/or motor-cognitive training [17–21]. Such technologies have therefore attracted significant interest as powerful tools to advance healthcare [22] and are recommended for health system improvements [23]. Well-designed and tailored exergame-based training effectively stimulates neuroplasticity and improves cognitive performance in individuals with mNCD [20, 24, 25]. It also optimizes resource allocation for home-based settings and remotely controlling, monitoring, supervising, and tailoring exercises [20, 26], with superior enjoyment and adherence compared with conventional exercises [27].

Exergame-enhanced interventions can be designed to provide superior adherence to relevant principles of behavior change, neuroscience, and exercise science, thereby contributing to the maintenance and improvement of overall health in ways that conventional forms of physical activity and therapy may not [21]. Given these advantages, exergame-based training is considered more promising than conventional exercise approaches [17–19, 21] and is emerging as the exercise modality that shows the greatest effects on general cognition and memory (on the basis of a meta-meta-analysis of 2,724 randomized controlled trials (RCTs)) [28]. Consequently, exergame-based training is recommended for health promotion and disease prevention across various populations and age groups [21], including those at risk for or with impairments in cognitive [20] or physical [29] functioning and emotional well-being [30], which is particularly relevant for mNCD that may affect all these domains.

Such positive effects of well-designed exergame-based training programs on cognitive performance [20] can theoretically be assumed to be associated with brain changes at the structural level [31]. However, a recent systematic review identified only one published empirical study that explored structural neural correlates of exergame-based training in individuals with mNCD, highlighting a knowledge gap in the current state of evidence [32]. This study observed that improvements in verbal memory were related to increases in dorsolateral PFC (dlPFC) volumes in eight mNCD patients, with higher exercise doses positively correlated with increases in gray matter volume (GMV) in the PFC and ACC [33].

Following recommendations to address the above-highlighted research gap [32], this study presents the first RCT exploring structural neural changes in the brain and their relationships to cognitive performance adaptations following the addition of co-designed, purpose-developed, and individually tailored exergame-based training to usual care.

## Methods

### Prior work

In the ‘Brain-IT’ project, we developed an individually tailored training program for secondary prevention of mNCD that is rooted in years of iterative co-design, purpose-development, and evaluation with continuous patient and public involvement [26]. The resulting ‘Brain-IT’ training concept serves as a guideline for implementing an *“exercise as medicine”* approach for the secondary prevention of mNCD by providing algorithmic decision trees for all aspects of the program structure, content, and individualized tailoring (i.e., personalization of training content and individualized progression of the exercises). It also includes detailed guidelines for its adaptation to other hardware to ensure broad applicability and scalability [34]. 

The project methodology [26] was a refined version of the Multidisciplinary Iterative Design of Exergames (MIDE) - Framework [35] and integrated the guidelines of the UK Medical Research Council for the development and evaluation of complex interventions [36]. In *phase 1*, we combined a comprehensive literature synthesis [26] with qualitative research including primary end-users (individuals with mNCD), secondary end-users (multidisciplinary healthcare professionals), and experts from the exergaming industry to collaboratively elaborate a set of design requirements for the exergame technology and training concept [37]. In *phase 2*, possible concepts were iteratively co-designed, developed, tested, and refined until an “acceptable” (feasible, usable, and safe, with high end-user acceptance) solution was achieved [38]. This process resulted in a novel intervention type targeting relevant mechanisms of action to alleviate the pathological state of mNCD by combining, for the first time, exergame-based multidomain training with biofeedback-guided resonance breathing training [34]. In *phase 3*, we confirmed the effectiveness of ‘Brain-IT’ training in improving global cognitive performance as well as immediate and delayed verbal recall. Compared with usual care, the training not only effectively slowed cognitive decline but resulted in 55% of participants showing clinically relevant improvements [34]. 

### Context

This manuscript is a part of a larger preregistered RCT evaluating the effectiveness of adding ‘Brain-IT’ training to usual care in older adults with mNCD compared with usual care alone, with global cognitive performance as the primary outcome. The results for all outcomes, except for magnetic resonance imaging (MRI)-derived outcomes, were published previously [34]. This paper is dedicated to GM and WM changes in response to ‘Brain-IT’ training, as defined as tertiary outcomes in the study protocol [39].

### Objectives

We aimed to explore structural brain changes following the addition of ‘Brain-IT’ training to usual care in individuals with mNCD compared with usual care alone. Furthermore, the neural correlates of previously reported cognitive performance adaptations were explored. Given these exploratory objectives, no prespecified hypotheses for MRI-derived outcomes were defined in the study protocol.

### Protocol, registration, and reporting

The study protocol for this RCT was published previously [39]. The study was prospectively registered at ClinicalTrials.gov prior to the start of patient recruitment (NCT05387057; date of registration: May 18, 2022). This manuscript adheres to the recommendations of the *“Consolidated Standards of Reporting Trials (CONSORT) Statement for Randomized Trials of Nonpharmacologic Treatments“* [40] (Supplementary File 1). For full reproducibility, please also refer to the study protocol [39].

### Explanation and choice of comparators

On the basis of the alignment of recommended treatment and management of individuals with mNCD in Switzerland with available clinical practice guidelines and consensus statements [41], we chose to compare the addition of ‘Brain-IT’ training to usual care versus usual care alone as a comparator [39]. This methodological choice aligns with the UK Medical Research Council Guidance [36].

### Overview of the trial design, participants, and interventions

A two-arm, prospective, parallel-group, single-blinded (i.e., outcome evaluator of pre- and postmeasurements blinded to group allocation) RCT with a 1:1 allocation ratio (i.e., intervention: control) on a sample of older adults with mNCD was conducted between May 2022 and February 2024. The study setup was multicentric (Zurich and St. Gallen) and national (Switzerland).

Individuals with mNCD were recruited between May 2022 and October 2023 in collaboration with (memory) clinics in the larger areas of Zurich and St. Gallen. After recruitment and providing written informed consent, participants were screened for eligibility (for a full list of eligibility criteria, see the study protocol [39]), and premeasurements were scheduled for all eligible participants. The key eligibility criteria included a clinical diagnosis of ‘mild neurocognitive disorder’ according to the International Classification of Diseases 11th Revision (ICD-XI) [42] or the latest Diagnostic and Statistical Manual of Mental Disorders 5th Edition (DSM-5) [43]; the absence of additional, clinically relevant (i.e., acute or symptomatic or both) neurological disorders (i.e., epilepsy, stroke, multiple sclerosis, Parkinson’s disease, brain tumors, or traumatic disorders of the nervous system); and being physically and cognitively capable of performing the intervention and assessment procedures. Information about the etiology of mNCD along with the clinical diagnosis and comorbidities was obtained from the collaborating recruitment partners. To ensure diversity, equity, and inclusion, all patients referred to us by the clinical recruitment partners were fully considered for participation in the study.

All participants with no contraindications to MRI had an MRI scan at University Hospital Zurich within two weeks prior (premeasurement) to starting and after completing the intervention (postmeasurement). All the measurements were led by PM and assisted by a trained investigator from our research team; both were trained in the application of measurement techniques and protocols. Pre- and postmeasurements were scheduled to take place at approximately the same time of day (± 2 h) for each participant. All participants were instructed verbally and in writing to follow a normal sleep routine the day before the experiment, to avoid intense physical activities and alcohol consumption within 24 h before measurements, and to refrain from coffee or caffeinated drinks as well as food consumption at least 2 h before measurements.

After completing the premeasurements, the participants were randomly allocated to either the intervention group or the control group and were instructed about their respective intervention procedures. The control group proceeded with usual care, as provided by the (memory) clinics where the patients were recruited. For participants in the intervention group, the exergame device was installed at their homes, followed by safety instructions and familiarization with the exergame training system. Consequently, participants started their twelve-week training intervention according to the ‘Brain-IT’ training concept in addition to usual care (see section *"Exergame training with biofeedback breathing – the ‘Brain-IT’ training concept"* for details).

After completing the twelve-week intervention period, postintervention measurements were performed for both groups. No monetary compensation was granted to the participants, but detailed feedback on individual performance as well as the study outcomes in general was provided at the end of the study. When possible, caregivers were actively involved in helping participants travel to the study centers for measurements and reminding them to adhere to the training plan.

### Exergame training with biofeedback breathing– the ‘Brain-IT’ training concept

The ‘Brain-IT’ training concept is a guideline for applying a holistic *“exercise as medicine”* approach for the secondary prevention of mNCD. This is achieved by (i) combining different exercise types to target various domains that are affected in individuals with mNCD, (ii) personalizing the training focus and exercise content on the basis of the clinical and neuropsychological assessment of a patient, and (iii) individually progressing the exercises on the basis of the physiological response and exergaming performance data. Specifically, the different exercise types entail a combination of multidomain (physical, motor, and cognitive) exercises delivered through serious exergames with biofeedback-guided resonance breathing– a neuromodulatory intervention to stimulate the vagus nerve and enhance self-regulatory abilities via central autonomous network regulation. Thereby, physical, motor, and cognitive functions as well as self-regulation are trained with a deficit-oriented focus on the neurocognitive domains of (1) learning and memory, (2) executive function, (3) complex attention, and (4) visuospatial skills [26, 34].

**Fig. 1:**
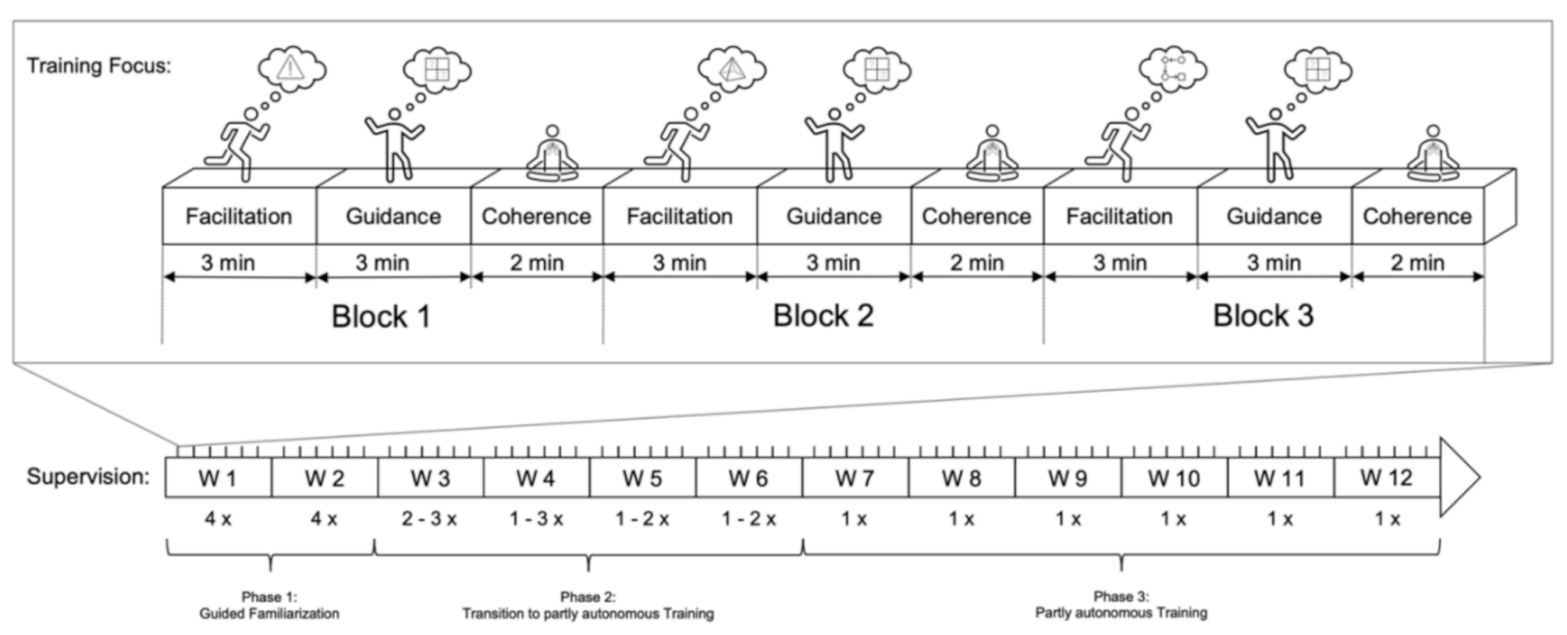
Overview of the structure of the ‘Brain-IT’ training concept

Figure 1 provides an overview of the structure of the ‘Brain-IT’ training concept, which standardizes the training characteristics (e.g., training frequency, intensity, and duration), as well as the structure and content of training, whereas the specific games to be played as well as their complexity and progression are to be determined following decision trees. Specifically, the games were individually assigned considering the participants’ disease etiology and baseline neuropsychological performance, with all participants starting at level 1 of the game demands in the first training session. Thereafter, either the games progressed or more difficult games were introduced as soon as (1) a plateau in performance was reached on predetermined game metrics, (2) a predetermined target score was reached on a predetermined game metric for progression to the next level, (3) the participant requested an increase in task demands, or (4) the staff supervising the participants deemed the increase in task demands feasible.

The exergame device and the specific games used within each of the defined neurocognitive domains are replaceable. To ensure replicability, the ‘Brain-IT’ training concept was planned and reported according to the Consensus on Exercise Reporting Template [44] and provides specific instructions on how to adapt the ‘Brain-IT’ training concept to other hardware and software solutions (see supplementary file 2 of [34]).

Each participant was instructed to train ≥ 5x/week for ≥ 24 min per session, resulting in a weekly training volume of ≥ 120 min. All training sessions took place at the participants’ homes. As per the ‘Brain-IT’ training concept [26], 19–24 training sessions were supervised by a designated study investigator who instructed and oversaw the participants’ use of the exergame device, ensured that safety protocols were followed [e.g., ensuring that there were no hard objects (e.g., couch table) within the potential drop zone, determining the appropriate level of stability support by walking sticks, handrails or similar), and ensured adherence and fidelity to the ‘Brain-IT’ training concept. All deviations from the ‘Brain-IT’ training concept were reported.

In this project, we used technology of Dividat AG (i.e., ‘Senso Flex’ (Dividat AG, Schindellegi, Switzerland; hardware: prototype version 2, software: version 22.4.0-360-gf9df00d5b), Polar (i.e., heart rate monitor (Polar M430) and sensor (Polar H10)), and Kubios (Kubios HRV Premium (Kubios Oy, Kuopio, Finland, version 3.4)) to implement the training. For more detail on how the specific technologies are used to implement our training concept, consider the full ‘Brain-IT’ training concept (supplementary file 2 of [34]). The exergame system automatically registered the data necessary for calculating and monitoring training adherence and progression, including the time spent interacting with each game and game performance, for both supervised and unsupervised sessions. Details on how these data were evaluated are reported in the section *“Data collection and statistics: study population & intervention delivery”*.

### Sample size

The sample size for the parent RCT was justified to obtain a sufficiently precise estimate of the treatment effect for the primary outcome (i.e., global cognitive performance) to allow sample size calculation for a subsequent full-scale confirmatory RCT [39] on the basis of Whitehead et al.’s (2016) method [45]. This substudy’s sample size was based on the number of participants for whom we had complete and evaluable MRI scan data.

### Randomization

To ensure allocation concealment, each participant was individually assigned either to the intervention or control group after completing the premeasurements. Randomization was performed by the study investigator assigned as the person responsible for supervision and correspondence with the respective study participant to ensure the blind keeping of outcome assessors. A variable block randomization model (i.e., block sizes = 4, 6, 8) with a 1:1 allocation ratio stratified by sex and institute (study center) was used.

### Blinding

The outcome assessors of the pre- and postmeasurements were blinded to group allocation (single blinding). To ensure the blinding and blind-keeping of all outcome assessors, detailed study-specific guidelines for all relevant procedures were established that were strictly followed by all involved study investigators. Blinding participants was not possible since usual care was used as a control intervention.

### Participant retention

Once a participant was included, a trained investigator was assigned as the person responsible for supervision and correspondence with the respective study participant and made all reasonable efforts to achieve the participant’s retention in the study, as explained in more detail in the study protocol [39] and publication of the parent RCT findings [34].

### Outcomes and analyses

#### General statistical procedures and interpretation of statistics

Data are reported as mean ± standard deviation for data fulfilling all the assumptions that would subsequently justify parametric statistical analyses. If these assumptions were not met, medians (interquartile ranges) were reported. Questionnaire scores were regarded as ordinal data. For all outcomes, descriptive statistics were computed first. The normal distribution of the data was checked via the Shapiro-Wilk test and visually confirmed via QQ plots. In line with the published study protocol [39], the level of significance was set to ɑ = 0.05 for between-group comparisons of baseline data, as well as checks of statistical assumptions.

This RCT was not adequately powered for neuroimaging outcomes, which, as per our study protocol [39], were tertiary outcomes aiming to explore (i.e., without prespecified hypotheses) the possible neural changes following the intervention in relation to adaptations in cognitive performance. These explorations aimed to obtain a first indication of whether ‘Brain-IT’ training impacts brain structures affected by the mNCD, thereby informing power calculations for subsequent hypothesis-driven studies. Power calculations for such studies rely mainly on an accurate estimate of the effect size and its variation [46], for which this study has been powered following Whitehead et al.’s (2016) method [45] (see Sect. 3.8 - sample size and published study protocol [39]), whereas other input variables for sample size determination (i.e., level of significance, targeted statistical power, anticipated drop-out rate, etc.) are usually determined in relation to the context and design of the study [46]. To accurately reflect these circumstances, to avoid missing potential clinically relevant effects that could be investigated further in subsequent hypothesis-driven studies due to the small sample size, and in line with recommendations [47], we decided for a level of significance of ɑ = 0.10 for all neuroimaging analyses related to intervention-related effects prior to any data analyses. In line with this approach and as recommended by Lee et al. (2014) [47], we focused our interpretation of the results more on effect size estimates and their confidence intervals (CIs) rather than statistical significance to provide a nuanced analysis of the results in relation to our exploratory objectives.

Statistical analysis was performed after data collection was completed. No interim, subgroup, or adjusted analyses were performed. To analyze intervention-related effects, we performed a modified intention-to-treat analysis (i.e., data from all randomized participants who completed pre- and postmeasurements, regardless of protocol adherence, were included in the statistical analyses).

For all analyses involving clearly prespecified regions or outcomes of interest, we report uncorrected *P* values. This decision reflects the targeted nature of these analyses and accounts for methodological limitations related to statistical power and sample size, as discussed in Section *“Strengths and limitations”*, as well as our approach to statistical interpretation focusing on effect size estimates outlined in the preceding section. In contrast, for fully exploratory whole-brain (voxel-wise) analyses, we report family-wise error corrected *P* values. This approach was necessary because the software used did not allow for region selection based on effect size thresholds, necessitating predetermined criteria for reporting of results (see Methods and Results Sections *“White matter structural integrity”*) for which we decided to use corrected *P* values to mitigate false positive findings.

#### Data collection and statistics: study population & intervention delivery

We collected data on age, sex, body mass index, years of education, physical activity behavior (i.e., measured with the German version of the International Physical Activity Questionnaire Short Form - short form [39]), and etiological subtype. Details on the delivery of the interventions (adherence to the ‘Brain-IT’ training and details on all usual care interventions, including medication intake (i.e., type and dosage of medication), as well as changes in medication during the study), were also assessed and reported elsewhere [34]. In short, participants who completed the training performed, on average, 71.5 ± 26.2 training sessions, resulting in an average training volume of 1689 ± 579 min over the 12-week intervention period. 100% of participants in the intervention group and 94% of participants in the control group reported that they received one or more structured or guided usual care activities(s) during study participation. These included mainly medication (100% of participants in the intervention group and 88% of participants in the control group), while between 0% and 25% of participants received nonpharmacological interventions such as physiotherapy, occupational therapy, medical training therapy, (computerized) cognitive training, psychiatric therapy or regular fitness training. There were no statistically significant between-group differences in the types of pharmacological or nonpharmacological interventions received.

For all demographic variables, between-group differences (i.e., intervention vs. control) were tested via an independent t test or the Mann-Whitney U test when the data were not normally distributed. Between-group differences in categorical variables were tested via Fisher’s exact test. To discover whether the between-group differences were substantive, Pearson’s r effect sizes were calculated and interpreted to be small (0.1 ≤ *r* < 0.3), moderate (0.3 ≤ *r* < 0.5) or large (*r* > 0.5) [48]. These statistical analyses were performed via R (The R Foundation; version 4.3.1 GUI 1.79 Big Sur Intel build) in line with RStudio (RStudio, Inc.; version 2023.12.1 + 402 (2023.12.1 + 402)).

#### Brain structure: data acquisition

Brain structure was recorded via MRI using a 3.0 Tesla Philips whole-body scanner (Philips Medical Systems, Best, and Netherlands) with a 32-channel head coil. All participants who had no contraindications to MRI, i.e., any MRI-incompatible metallic parts in the body, metallic or electronic implants (e.g., heart or brain pacemakers and cochlear implants), and strong claustrophobia, were measured. All participants were instructed to avoid head movements during the measurements.

Data were collected according to the Canadian Dementia Imaging Protocol [49] for comparability with other studies. CDIP was developed to harmonize MRI acquisitions in the context of studying primary and secondary causes of morbidity of neurodegeneration in a wide range of neurological pathologies related to aging [49]. All the measurements were conducted using the same scanner with identical equipment, setup, and measurement parameters. The following imaging sequences of the core CDIP [49] were acquired:

- 3D isotropic T_1_-weighted (T_1_w) scan (duration = 6.5 min) for assessing fine anatomical detail and brain atrophy (voxel size = 1.0 × 1.0 × 1.0 mm^3^) with an acceleration factor of 2 (TFE-Sense: 2), 180 slices, field of view: 256 × 248 mm², flip angle: 9°, repetition time: 7.3 ms and echo time: 3.3 ms.
- Diffusion tensor imaging (DTI; duration = 6 min) for the assessment of WM microstructural integrity and connectivity, a voxel resolution of 2.0 × 2.0 × 2.0 mm^3^, 30 uniformly distributed directions with b = 1000 s/mm^2^ (EPI-Sense 2–32 directions; we use the vendor-provided directions set), and an acceleration factor of 2, field of view: 240 × 240 mm², 70 contiguous transversal slices, flip angle: 90°, repetition time: 13.44 s and echo time: 102 ms.

### Brain structure: data analysis and statistical procedures

#### Gray and white matter volumes

T_1_-weighted scan data quality was assessed with segmentation and sample homogeneity tools from the Computational Anatomy Toolbox for SPM (see Supplementary File 2). Processing and volumetric segmentation of the T_1_w morphological data were performed via the longitudinal *recon* pipeline of the FreeSurfer software package (version 7.4.1 with default parameters) [50, 51], as described previously [52]. GMVs and WM volumes (WMVs) were determined for the following regions of interest (ROIs): total brain (i.e., total brain volume without ventricles [brainsegvolnotvent] from the aseg file), hippocampus, dlPFC, PFC, ACC, and thalamus. We combined the following regions to obtain the defined ROIs of the dlPFC, PFC, and ACC because they are not directly extracted via the recon all pipeline: dlPFC = sum of the pars opercularis, pars triangularis, caudal middle frontal, and rostral middle frontal; ACC = rostral and caudal anterior cingulate cortices; PFC = frontal pole + superior frontal + rostral middle frontal + rostral anterior cingulate + pars triangularis + pars orbitalis + pars opercularis + medial orbitofrontal + lateral orbitofrontal + caudal middle frontal + caudal anterior cingulate. All regions were defined via the Desikan et al. [53] atlas. In accordance with common practice in between-participant comparisons of regional brain estimates, extracted volume estimates were normalized with total intracranial volume to account for interindividual differences in head size [54, 55].

For GMVs and WMVs, the assumption of homogeneity of variance was checked via Levene’s test. If all assumptions for an analysis of covariance (ANCOVA) were met, an ANCOVA with the premeasurement value as a covariate for the predicting group factor, the postmeasurement value as the outcome variable, and age, sex and the etiological subtype (i.e., mNCD due to AD, mild frontotemporal NCD, mNCD with Lewy bodies, or mild vascular NCD) as additional covariates was performed [48]. If not all assumptions were met, Quade’s nonparametric ANCOVA was used. To quantify the magnitude of the effects, partial eta-squared (η^2^_p_) effect sizes were calculated and interpreted to be small (0.01 ≤ η^2^_p_ < 0.06), moderate (0.06 ≤ η^2^_p_ < 0.14) or large (η^2^_p_ ≥ 0.14) [46]. These statistical analyses were performed via R (The R Foundation; version 4.3.1 GUI 1.79 Big Sur Intel build) in line with RStudio (RStudio, Inc.; version 2023.12.1 + 402).

#### White matter structural integrity

DTI data were preprocessed via the pipeline created by Stämpfli et al. [56], extracting values for fractional anisotropy (FA), mean diffusivity (MD) and radial diffusivity (RD) [56–59]. The pipeline performs brain extraction (“bet”), motion and eddy current correction (“eddy”) and tensor fitting (“dtifit”) with tools from the FSL software package. Using the anatomical T_1_w data, susceptibility-induced distortion correction was performed with the “bdp” tool from the BrainSuite package. Quality control was performed via the ‘diffQC’ [web link (WL) 1] package, which is based on MRtrix3 [WL 2], and checks for completeness - data for pre- and postmeasurement present.

We performed a tract-based spatial statistical (TBSS) analysis to study WM changes at the voxel-to-voxel (whole-brain) level. For this analysis, we used the recommended default settings, registering all FA images to the FMRIB58 template and extracted a skeleton map containing the main common tracts extracted from the averaged FA of all participants using a default threshold of 0.2 [60]. This map was then used to project the FA data of each dataset onto this mean skeleton. RD and MD data are preprocessed through the ‘tbss_non_FA’ command, which uses the outcomes of FA preprocessing to analogously process the other DTI measures [WL 3]. The TBSS analysis is based on a general linear model and was performed via FSL’s ‘randomize’ tool [61] with 5000 permutations calculating threshold-free cluster enhancement (TFCE) [62] with and without familywise error (FWE) correction.

A repeated measure analysis of variance with associated one-tailed t-tests were performed. The contrast examining the main effect of intervention (post-to-pre change between the intervention and control group) was calculated by computing one tailed t-test of the datasets gained by the subtraction of the pre from post dataset of each participant (referred to as ∆ (post-to-pre) datasets) using “fslmaths”. These contrasts were computed testing for positive and negative differences in FA, RD and MD. We included age, sex and the etiological subtype as covariates in the model reporting both the FWE-corrected values and the overlapping uncorrected values. The latter are masked with the significant areas with the FWE correction, therefore reporting uncorrected data only in voxels that are significant in the corrected contrast. The voxel amount and peak *P* values were extracted from the output of the TBSS analysis segmented according to the ‘JHU-ICBM-DTI-81’ atlas [WL 4]. For the calculation of the median, interquartile ranges, and effect sizes, we pooled the data of participants in the respective groups, interventions, and controls; then, we aggregated all points of the skeletonized dataset in an area of the ‘JHU-ICBM-DTI-81’ atlas [WL 4]; and compared the groups. We chose to report on Cliff’s δ due to its robustness with a non-normal distribution of datasets. We decided to limit comparisons to potentially relevant areas, defined as areas that reached statistical significance in the TBSS analysis. These calculations were performed using Matlab (version: R2024a).

#### Neural correlates of cognitive performance improvements

To explore the neural correlates of the previously reported cognitive performance adaptations in response to the interventions [34], we ran one-sided bivariate correlation analyses to investigate the relationships between changes (i.e., ∆ post-to-pre) in (i) brain structure and (ii) cognitive performance. These analyses included data from all participants (both the intervention and control groups). We predetermined limiting the analysis to outcomes that fulfilled the criteria of statistical significance in the main effect of intervention to focus our inquiry on potentially relevant areas and curtail the effect of spurious random effects. These outcomes included (i) global cognitive performance, (ii) immediate verbal recall, and (iii) delayed verbal recall for neuropsychological assessments. Global cognitive performance was assessed using the validated German version [63] of the Quick mild cognitive impairment screen (Qmci) [64, 65]. While originally developed as a screening tool, it was shown to be sensitive for changes in cognitive performance over time [66] with a clinically meaningful change defined as a change in ≥ 3 points in the total score [39]. Immediate and delayed verbal recall were assessed using the validated German version of the subtests ‘logical memory’ of the Wechsler Memory Scale - fourth edition [67, 68]. All cognitive assessments were administered and evaluated according to published guidelines [65, 67]. For completeness, we also repeated our analyses on effects of the intervention on these cognitive endpoints in the subsample of participants with complete MRI data available and analyzed in the context of this study using an ANCOVA (same methods as for GMs and WMVs). Depending on the distribution of the data, either Pearson (parametric statistical analyses) or Spearman (nonparametric statistical analyses) correlation analyses were conducted. *P* values and correlation coefficients (Pearson r (r) or Spearman rho (ρ)) including 90% CI were calculated (using bootstrap ('boot' function in R) with 5000 resamples and otherwise default settings for nonparametric analyses)) [48]. Spearman ρ correlation coefficients were interpreted as weak ( ρ < 0.3), moderate (ρ  = 0.3–0.5), or strong (ρ > 0.5), identical to Pearson r [48]. We decided against applying Bonferroni correction for multiple comparisons in these analyses considering the exploratory nature of our analyses and considering that applying the Bonferroni correction in such a context may hinder the detection of clinically relevant associations [69].

### Important modifications to the study protocol

Recruitment was extended by six weeks. This allowed us to stop recruitment only once we had complete data on the primary outcome for the planned minimum sample size of 34 participants (see Section "Sample size"). Furthermore, there are four important departures from the published study protocol in data analysis (see Section "Outcomes and Analyses" for more detail) that must be noted. **First**, the ROI definitions in the volumetric analyses of the T_1_w scan data were refined so that all the ROIs were derived from the Desikan atlas. This allowed the use of the unaltered output of the “recon-all” pipeline. **Second**, the thalamus was added as a ROI prior to any analyses to better capture most relevant substructures of the central autonomous network, which was of particular interest to us given the HRV-BF training [70, 71]. **Third**, we refined the DTI analysis starting with the preprocessing pipeline, which was optimized to increase the signal-to-noise ratio. Furthermore, rather than running a tractography and TBSS analyses in parallel, we decided to drop tractography due to (i) resource limitations and (ii) limited comparability to other research and focused on the whole-brain TBSS analysis approach, in accordance with what has been defined in the study protocol [39]. This allows for better comparability with the literature by using a well-established WM atlas (JHU-ICBM-DTI-81; [WL 4]). The **fourth** modification to the published study protocol was the additional inclusion of analyses of neural correlates of previously reported improvements in cognitive performance. This addition is justified on the basis of the recommendations of a recent systematic review highlighting that there is a research gap in this regard in exergame-based studies in older adults with or without mNCD [32] and to better reflect the objective defined in the study protocol *“to explore the possible underlying neural changes in training in relation to adaptations in cognitive performance”* [39].

## Results

**Fig. 2:**
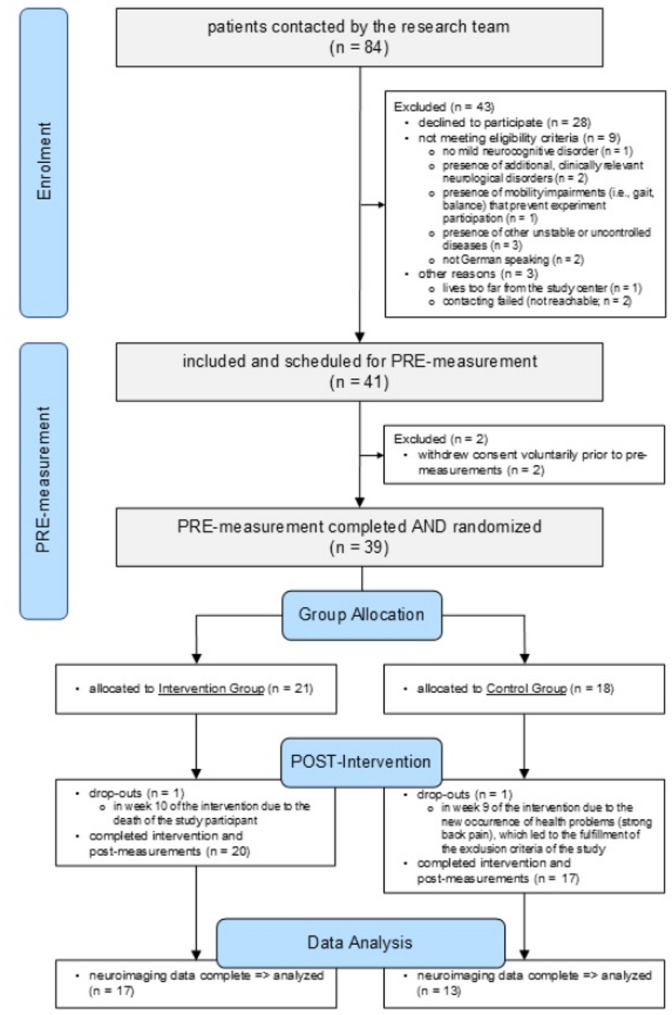
Summary of the participants’ flow throughout the study

A summary of the participant flow is illustrated in Fig. 2. Details on the delivery of the interventions were previously reported [34]. For the present structural neuroimaging analyses, we had complete datasets from 30 study participants (72.0 ± 8.6 years; 27% females). Data from all 30 study participants were included for volumetric analyses of the T_1_w data. For DTI analyses, visual inspection of the quality control (WL 1) output led to the exclusion of 5 datasets due to signal dropouts attributed to head motion artifacts.

### Baseline data

Table 1 summarizes the demographic characteristics of the participants. 87% of participants had a biomarker-supported characterization of the etiology of mNCD, mostly Alzheimer’s etiology (62%, based on the Aβ42/40 ratio in cerebrospinal fluid). A small effect size was observed for a higher BMI in the intervention group, although the means of both groups fell within the range of a ‘healthy’ BMI. However, no statistically significant between-group differences were found.

**Table 1:** Demographic characteristics of the study population

	Group: ‘Brain-IT’ Training (*n* = 17)	Group: Usual Care (*n* = 13)	Between-Group Difference		
			test statistics^(1)^	*P* Value^(2)^	|effect size|^(3)^
Age [years]	73.9 ± 7.5	69.5 ± 9.7	t = 1.38	0.18	*r* = 0.28
Sex [% females]	29.4	23.1	N/A	1.00	OR = 1.37
Body mass index [kg·m^-2^]	24.9 ± 2.3	23.1 ± 3.1	t = 1.78	0.09	*r* = 0.36
Years of education [years]	16.0 ± 3.8	14.8 ± 3.7	t = 0.839	0.41	*r* = 0.16
IPAQ-SF [MET·week^-1^]	2226 (2334)	1386 (1502)	W = 148	0.12	ρ = 0.28
Etiology of mNCD					
mNCD due to Alzheimer’s Disease	*n* = 9 (52.9%)	*n* = 7 (53.8%)	N/A	1.00	OR = 0.97
mild frontotemporal NCD	*n* = 3 (17.6%)	*n* = 0 (0%)	N/A	0.24	OR = ∞
mild vascular NCD	*n* = 2 (11.8%)	*n* = 4 (30.8%)	N/A	0.36	OR = 0.31
mNCD with Lewy Bodies	*n* = 0 (0.0%)	*n* = 1 (7.7%)	N/A	0.43	OR = 0.00
unclear/not yet determined	*n* = 3 (17.6%)	*n* = 1 (7.7%)	N/A	0.43	OR = 0.00

Data are reported as the mean ± standard deviation for continuous parametric data and median (interquartile range) for continuous nonparametric data

(1) t-statistics for the between-group differences tested with an independent t-test or Mann-Whitney U test in case the data are not normally distributed

(2) *P* values for the between-group differences tested with an independent t-test or Mann-Whitney U test in case the data are not normally distributed, or Fisher’s exact test for categorical variables

(3) effect size estimates for the between-group differences tested with an independent t-test ( = > effect size Pearson r) or Mann-Whitney U test ( = > effect size Spearman rho (ρ)) in case the data are not normally distributed, or Fisher’s exact test for categorical variables ( = > odds ratio)

Abbreviations: mild neurocognitive disorder; IPAQ-SF, International Physical Activity Questionnaire Short Form - short form (IPAQ-SF); IQR, interquartile rage; MET, metabolic equivalent of task; n, sample size; OR, odds ratio; SD, Standard Deviation

- Cognitive endpoints.

In the subsample of participants with complete MRI data available, we observed that the intervention group improved their score in global cognitive performance (primary outcome of parent study) from 58.9 ± 16.4 points at premeasurements to 62.1 ± 14.4 points at postmeasurements, while the control group showed a decline from 51.6 ± 15.9 points to 48.7 ± 16.2 points. There was a statistically significant effect with a large effect size (F [1, 26] = 4.97, *P* = 0.03, η^2^_p_ [CI_90%_] = 0.161 [0.006, 0.356]) in favor of the intervention group. For immediate and delayed verbal recall (secondary outcomes of parent study), we also observed statistically significant effects with moderate to large effect sizes (F [1, 26] = 4.35, *P* = 0.04, η^2^_p_ [CI_90%_] = 0.131 [0.001, 0.325], and F [1, 26] = 5.67, *P* = 0.025, η^2^_p_ [CI_90%_] = 0.179 [0.013, 0.374]) in favor of the intervention group.

- GMV and WMV.

The results for the GMVs and WMVs are detailed in Table 2. The quality checks revealed that all the participants were in the good to optimal range of the quality measure (Supplementary File 2).

**Table 2:** Statistics for Gray and white matter volumetry

Outcome:	Check of Assumptions and Type of Analysis:		Group: ‘Brain-IT’ Training			Group: Usual Care			ANCOVA Statistics:		
			PRE- measurement	POST- measurement	sample	PRE- measurement	POST- measurement	sample			
	All assumption for parametric analysis met?	type of analysis	mean ± SD or median (IQR)	mean ± SD or median (IQR)	*n*	mean ± SD or median (IQR)	mean ± SD or median (IQR)	*n*	*P* Value	F Value	η^2^_*p*_ [90% CI]
Part 1 - Gray Matter Volumes											
*1.1 Total Brain (without Ventricles)*											
total (both hemispheres) [mm^3^]	✓	parametric	1’010’420 (75’193)	1’008’290 (85’059)	17	1’021’364 (110’784)	1’016’852 (110’532)	13	0.15	2.18	**0.075 [0**,** 0.254]**
*1.2 Hippocampus*											
left hemisphere [normalized; % TIV]	✓	parametric	0.19 ± 0.05	0.20 ± 0.05	17	0.20 ± 0.04	0.20 ± 0.04	13	0.14	2.36	**0.080 [0**,** 0.261]**
right hemisphere [normalized; % TIV]	✓	parametric	0.21 ± 0.05	0.21 ± 0.05	17	0.21 ± 0.05	0.21 ± 0.05	13	**0.08**	3.30	**0.109 [0**,** 0.296]**
total [normalized; % TIV]	✓	parametric	0.40 ± 0.09	0.41 ± 0.09	17	0.41 ± 0.09	0.41 ± 0.09	13	**0.07**	3.48	**0.114 [0**,** 0.303]**
*1.3 Dorsolateral Prefrontal Cortex*											
left hemisphere [normalized; % TIV]	✓	parametric	1.52 ± 0.16	1.54 ± 0.15	17	1.61 ± 0.18	1.62 ± 0.19	13	0.39	0.78	0.028 [0, 0.180]
right hemisphere [normalized; % TIV]	✓	parametric	1.51 ± 0.16	1.54 ± 0.14	17	1.57 ± 0.18	1.57 ± 0.19	13	0.44	0.62	0.023 [0, 0.169]
total [normalized; % TIV]	✓	parametric	3.04 ± 0.29	3.08 ± 0.29	17	3.18 ± 0.33	3.19 ± 0.35	13	0.41	0.70	0.025 [0, 0.175]
*1.4 Prefrontal Cortex*											
left hemisphere [normalized; % TIV]	✓	parametric	4.25 ± 0.43	4.29 ± 0.41	17	4.42 ± 0.34	4.41 ± 0.38	13	0.19	1.80	**0.062 [0**,** 0.237]**
right hemisphere [normalized; % TIV]	✓	parametric	4.21 ± 0.42	4.25 ± 0.40	17	4.34 ± 0.35	4.35 ± 0.39	13	0.56	0.36	0.013 [0, 0.145]
total [normalized; % TIV]	✓	parametric	8.46 ± 0.84	8.54 ± 0.80	17	8.76 ± 0.68	8.76 ± 0.76	13	0.33	1.01	0.036 [0, 0.195]
*1.5 Anterior Cingulate Cortex*											
left hemisphere [normalized; % TIV]	✓	parametric	0.23 ± 0.06	0.23 ± 0.06	17	0.26 ± 0.05	0.25 ± 0.05	13	0.19	1.79	**0.062 [0**,** 0.236]**
right hemisphere [normalized; % TIV]	✓	parametric	0.21 ± 0.04	0.21 ± 0.05	17	0.23 ± 0.03	0.23 ± 0.03	13	0.29	1.16	0.041 [0, 0.204]
total [normalized; % TIV]	✓	parametric	0.44 ± 0.09	0.44 ± 0.08	17	0.49 ± 0.06	0.48 ± 0.05	13	0.94	0.01	0.000 [0, 0.014]
*1.6 Thalamus*											
left hemisphere [normalized; % TIV]	x	nonparametric	0.38 (0.06)	0.40 (0.06)	17	0.38 (0.05)	0.39 (0.07)	13	**0.02**	5.60	**0.172 [0.012**,** 0.364]**
right hemisphere [normalized; % TIV]	x	nonparametric	0.38 (0.05)	0.38 (0.05)	17	0.38 (0.04)	0.38 (0.05)	13	0.12	2.65	0.089 [0, 0.273]
total [normalized; % TIV]	x	nonparametric	0.74 (0.11)	0.75 (0.12)	17	0.79 (0.09)	0.77 (0.10)	13	0.82	0.05	0.002 [0, 0.081]
Part 2– White Matter Volumes											
*1.1 Total Brain (without Ventricles)*											
total (both hemispheres) [mm^3^]	x	nonparametric	51’545 (12’846)	52’397 (11’384)	17	48’981 (29’434)	48’749 (30’311)	13	0.17	2.019	**0.070 [0**,** 0.247]**
*1.2 Para-Hippocampus*											
left hemisphere [normalized; % TIV]	x	nonparametric	0.09 (0.00)	0.09 (0.00)	17	0.08 (0.02)	0.08 (0.02)	13	0.20	1.69	0.058 [0, 0.232]
right hemisphere [normalized; % TIV]	x	nonparametric	0.08 (0.02)	0.09 (0.02)	17	0.08 (0.02)	0.08 (0.03)	13	0.98	0.00	0.000 [0, 0.000]
total [normalized; % TIV]	x	nonparametric	0.18 (0.03)	0.18 (0.03)	17	0.18 (0.04)	0.16 (0.04)	13	0.37	0.85	0.031 [0, 0.185]
*1.3 Dorsolateral Prefrontal Cortex*											
left hemisphere [normalized; % TIV]	✓	parametric	1.42 ± 0.13	1.42 ± 0.12	17	1.53 ± 0.16	1.53 ± 0.15	13	0.58	0.31	0.011 [0, 0.140]
right hemisphere [normalized; % TIV]	✓	parametric	1.42 ± 0.14	1.42 ± 0.13	17	1.48 ± 0.12	1.48 ± 0.13	13	0.58	0.32	0.012 [0, 0.140]
total [normalized; % TIV]	✓	parametric	2.84 ± 0.25	2.84 ± 0.24	17	3.01 ± 0.26	3.00 ± 0.25	13	0.93	0.01	0.000 [0, 0.026]
*1.4 Prefrontal Cortex*											
left hemisphere [normalized; % TIV]	✓	parametric	4.03 ± 0.29	4.02 ± 0.31	17	4.24 ± 0.34	4.22 ± 0.32	13	0.55	0.36	0.013 [0, 0.145]
right hemisphere [normalized; % TIV]	✓	parametric	4.00 ± 0.29	4.00 ± 0.32	17	4.15 ± 0.34	4.15 ± 0.36	13	0.69	0.16	0.006 [0, 0.118]
total [normalized; % TIV]	✓	parametric	8.02 ± 0.57	8.03 ± 0.61	17	8.39 ± 0.67	8.37 ± 0.66	13	0.54	0.37	0.014 [0, 0.147]
*1.5 Anterior Cingulate Cortex*											
left hemisphere [normalized; % TIV]	✓	parametric	0.31 ± 0.05	0.31 ± 0.05	17	0.32 ± 0.05	0.32 ± 0.05	13	0.21	1.64	0.057 [0, 0.229]
right hemisphere [normalized; % TIV]	✓	parametric	0.26 ± 0.03	0.27 ± 0.03	17	0.28 ± 0.03	0.28 ± 0.03	13	0.35	0.90	0.032 [0, 0.188]
total [normalized; % TIV]	✓	parametric	0.57 ± 0.06	0.58 ± 0.06	17	0.60 ± 0.05	0.59 ± 0.05	13	**0.02**	6.21	**0.187 [0.018**,** 0.379]**

Normality distribution of data was checked using the Shapiro-Wilk test and Q-Q-plots. The level of significance was set to ɑ = 0.10 (two-sided, uncorrected). The assumption of homogeneity of variance was checked using Levene’s test. In case all assumptions for analysis of covariance (ANCOVA) were met, effects of the addition of the ‘Brain-IT’ training concept to usual care as compared to usual care were analyzed using an ANCOVA with the premeasurement value as covariate for the predicting group factor and the postmeasurement value as outcome variable. In case not all assumptions were met, Quade’s nonparametric ANCOVA was used. To discover whether effects are substantive, partial eta-squared (η^2^_p_) effect sizes were calculated. Effect sizes were interpreted to be small (0.01 ≤ η^2^_p_ < 0.06), medium (0.06 ≤ η^2^_p_ < 0.14) or large (η^2^_p_ > 0.14)

bold font = statistically significant results AND/OR moderate to large effect size

Abbreviations: SD, standard deviation; IQR, interquartile range; n, sample size; ANCOVA, analysis of covariance; η^2^_p_ [90% CI], partial eta-squared [90% confidence interval]; TIV, total intracranial volume

For **GMV**, we observed moderate to large effects in the right hippocampus, total hippocampus, and left thalamus in favor of the intervention group, which were statistically significant. Specifically, a moderate effect size for the GMVs of the total hippocampus (η^2^_p_ [90% CI] = 0.114 [0, 0.303]) and right hippocampus (η^2^_p_ [90% CI] = 0.109 [0, 0.296]) and large effect sizes for the GMV in the left thalamus (η^2^_p_ [90% CI] = 0.172 [0.012, 0.364]), all normalized for TIV, were observed. We also observed moderate effect sizes in favor of the intervention group in the following ROIs: total brain (without ventricles; η^2^_p_ [90% CI] = 0.075 [0, 0.254]) and normalized GMVs of the left hippocampus (η^2^_p_ [90% CI] = 0.080 [0, 0.261]), left PFC (η^2^_p_ [90% CI] = 0.062 [0, 0.237]), left ACC (η^2^_p_ [90% CI] = 0.062 [0, 0.236]), and right thalamus (η^2^_p_ [90% CI] = 0.089 [0, 0.273]). These effects, however, were not statistically significant. No relevant effects were observed for the remaining ROIs.

For **WMV**, we observed a large effect in favor of the intervention group in the total ACC (η^2^_p_ [90% CI] = 0.187 [0.018, 0.379]), normalized for TIV, which was statistically significant. No relevant effects were observed for the remaining ROIs.

The results on **brain correlates** to the observed improvements in cognitive performance are displayed in Table 3, with the corresponding plots in Supplementary File 3. The improvements in verbal delayed recall were positively correlated with the changes in GMVs of the total hippocampus (total and right) and left thalamus, all with weak correlation coefficients (i.e., 0.280 [-0.067 to 0.556], 0.263 [-0.075 to 0.546], and 0.279 [-0.065 to 0.541], respectively). The remaining correlation analyses revealed no statistically significant observations.

**Table 3:** Neural correlates in Gray and white matter volume changes to observed cognitive performance improvements

Region of Interest [normalized; % total intracranial volume]	Statistical Results		
	Correlation Coefficient (i.e., Pearson *r* (*r*) or Spearman rho (ρ)) [90% Confidence Interval]	*P* value	Sample size
*Neural Correlates to Global Cognitive Performance Adaptations*:			
∆ GMV of hippocampus - total	*r* = -0.083 [-0.318 to 1.000]	0.67	30
∆ GMV of hippocampus - right	*r* = -0.104 [-0.338 to 1.000]	0.71	30
∆ GMV of thalamus - left	ρ = 0.169 [-0.131 to 0.444]	0.19	30
∆ WMV of anterior cingulate cortex - total	*r* = -0.034 [-0.274 to 1.000]	0.57	30
*Neural Correlates to Immediate Verbal Recall Adaptations*:			
∆ GMV of hippocampus - total	*r* = 0.080 [-0.169 to 1.000]	0.34	29^(1)^
∆ GMV of hippocampus - right	*r* = 0.096 [-0.154 to 1.000]	0.31	29^(1)^
∆ GMV of thalamus - left	ρ = 0.238 [-0.102 to 0.538]	0.11	29^(1)^
∆ WMV of anterior cingulate cortex - total	*r* = -0.230 [-1.000 to 0.017]	0.12	29^(1)^
*Neural Correlates to Delayed Verbal Recall Adaptations*:			
∆ GMV of hippocampus - total	ρ = 0.280 [-0.067 to 0.556]	0.07	29^(1)^
∆ GMV of hippocampus - right	ρ = 0.263 [-0.075 to 0.546]	0.08	29^(1)^
∆ GMV of thalamus - left	ρ = 0.279 [-0.065 to 0.541]	0.07	29^(1)^
∆ WMV of anterior cingulate cortex - total	*r* = 0.096 [-0.285 to 0.465]	0.31	29^(1)^

(1) Missing data from one study participants for cognitive performance assessment because measurement was aborted due to emotional breakdown

Abbreviations: GMV, gray matter volume; WMV, white matter volume

- White matter integrity.

The results from the analysis of DTI data are reported in Table 4.

**Table 4:** Table illustrating the results on white matter structural integrity

White Matter Tract	Statistical Results					
	number of significant voxels		Group: ‘Brain-IT’ Training - Median (IQR)	Group: Usual Care - Median (IQR)	Corrected peak p-values	Effect size Cliff’s δ [90% Confidence Interval]
	corrected	uncorrected				
*Group Contrast (n = 25): Fractional Anisotropy - Group* *‘Brain-IT’ Training > Usual Care (post-to-pre)*:						
Body of corpus callosum	47	10	1.16E^-03^(9.44E^-02^)	-1.66E^-03^(8.52E^-02^)	0.08	0.019 [0.011, 0.027]
Splenium of corpus callosum	301	149	5.44E^-03^(1.06E^-01^)	-8.99E^-03^(9.93E^-02^)	0.08	0.100 [0.091, 0.109]
Right Retrolenticular part of internal capsule	97	34	4.06E^-03^(8.90E^-02^)	-7.27E^-03^(8.48E^-02^)	0.06	0.082 [0.064, 0.099]
Right Superior corona radiata	15	1	4.92E^-03^(5.63E^-02^)	2.59E^-03^(5.29E^-02^)	0.09	0.027 [0.015, 0.039]
Right Posterior corona radiata	158	12	3.74E^-03^(7.39E^-02^)	-5.14E^-03^(6.89E^-02^)	0.08	0.088 [0.070, 0.106]
Right Posterior thalamic radiation (includes optic radiation)	292	101	3.15E^-03^(1.08E^-01^)	-1.30E^-02^(9.96E^-02^)	0.06	0.120 [0.106, 0.134]
Right Sagittal stratum (includes inferior longitudinal fasciculus and inferior fronto-occipital fasciculus)	60	47	3.05E^-04^(9.45E^-02^)	-3.94E^-03^(8.85E^-02^)	0.07	0.025 [0.007, 0.043]
Right Superior longitudinal fasciculus	288	66	5.45E^-03^(7.09E^-02^)	-6.31E^-03^(6.50E^-02^)	0.06	0.121 [0.110, 0.133]
Right Tapetum	6	1	6.81E^-03^(1.11E^-01^)	-6.76E^-03^(1.11E^-01^)	0.07	0.038 [-0.039, 0.115]
*Group Contrast (n = 25): Radial Diffusivity - Group* *‘Brain-IT’ Training < Usual Care (post-to-pre)*:						
Body of corpus callosum	109	70	-1.13E^-06^(1.54E^-04^)	5.00E^-06^(1.33E^-04^)	0.07	-0.034 [-0.043, -0.026]
Splenium of corpus callosum	225	164	-1.10E^-05^(2.17E^-04^)	2.32E^-05^(2.08E^-04^)	0.07	-0.122 [-0.131, -0.112]
Left Posterior corona radiata	5	0	-1.97E^-06^(1.16E^-04^)	5.56E^-06^(1.10E^-04^)	0.10	-0.048 [-0.067, -0.030]
Left Posterior thalamic radiation (includes optic radiation)	1	1	-5.91E^-06^(1.95E^-04^)	5.15E^-06^(1.68E^-04^)	0.10	-0.043 [-0.057, -0.029]
Left Tapetum	3	1	1.41E^-05^(3.38E^-04^)	1.16E^-04^(3.53E^-04^)	0.10	-0.283 [-0.446, -0.103]

Illustrated are all the JHU-ICBM-81 atlas regions that showed significance (ɑ = 0.1) in the threshold-free cluster enhancement familywise error corrected p-values, in the ∆ (post-pre recordings) of the Intervention > Control for fractional anisotropy contrast and Intervention < Control for radial diffusivity contrast. The uncorrected values refer to the voxel that showed significance, collocated with the voxel that trended after familywise error correction

Abbreviations: IQR, interquartile range; n, sample size;

**Fig. 3:**
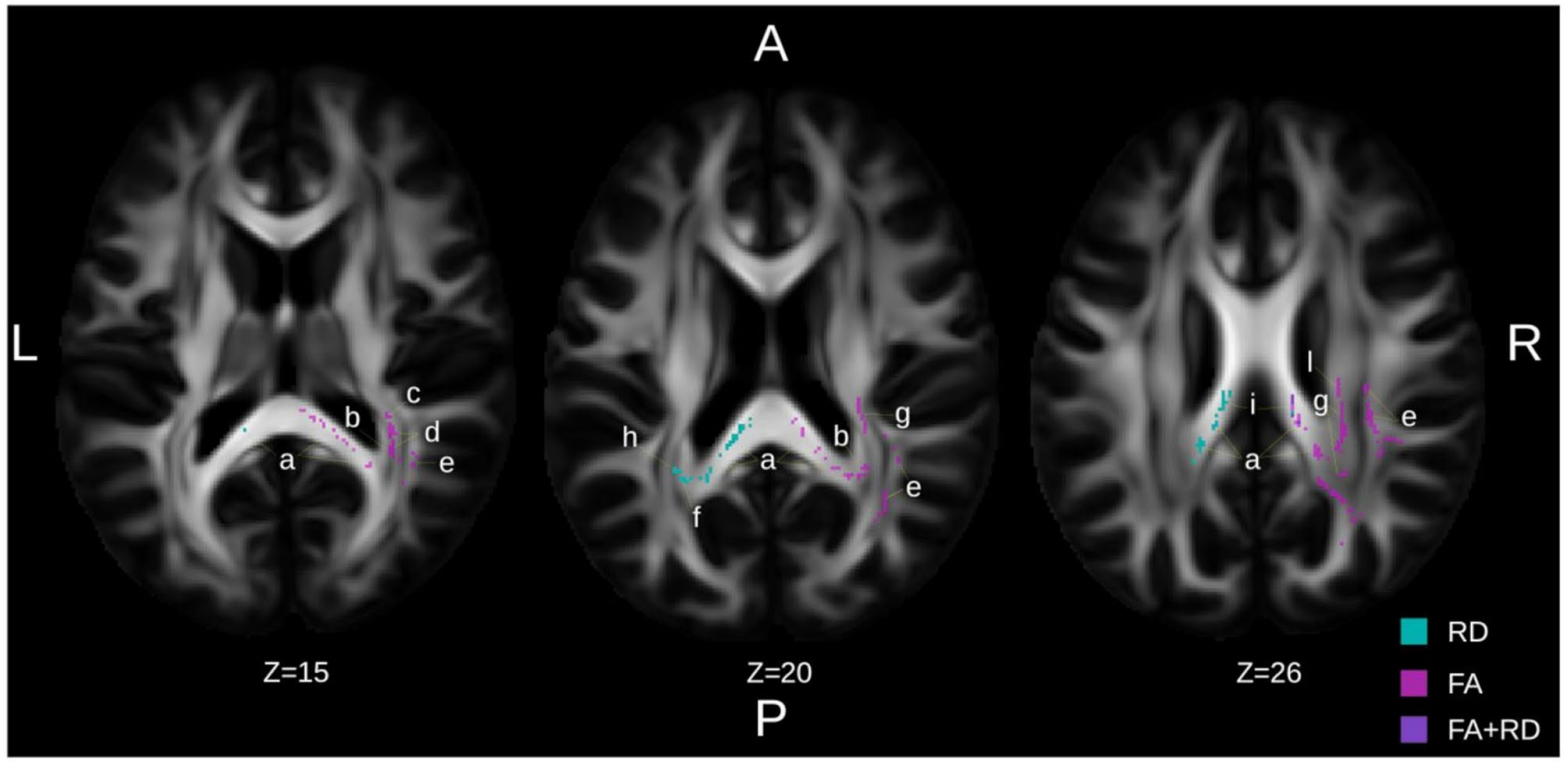
Results from tract-based spatial statistical analysis to study white matter changes at voxel-to-voxel (whole-brain) level. The figure illustrates statistically significant voxels in the ∆ (post-to-pre) recordings of the Intervention >Control for fractional anisotropy and Intervention < Control in slices selected to illustrate most of the involved JHU-ICBM-81 atlas regions. To note is the contralateral distribution in the Corpus Callosum (CC) of the FA and RD measures, as well as the overlap in the body of CC. The unlabeled regions posterior to the right posterior corona radiata in slice Z = 26 are “Unclassified” in the JHU-ICBM-81, the Talairach Daemon 10.1002/1097-0193(200007)10:3 classifies this area as WM in the right parietal lobe. JHU-ICBM-DTI-81 atlas regions: **a)** Splenium of CC, **b)** Right tapetum, **c)** Right retrolenticular part of internal capsule, **d)** Right posterior thalamic radiation (include optic radiation), **e)** Right superior longitudinal fasciculus **f)** Left tapetum **g)** Right posterior corona radiata **h)** Left posterior corona radiata **i)** Body of CC

For FA, statistically significant tracts for the main effect of intervention (i.e., ∆ (post-to-pre) dataset) with peak values of *p* = 0.06 in the right superior corona radiata, the right superior longitudinal fasciculus and the left optic radiation (FWE corrected *p*_*TFCE*_ = 0.09; JHU-ICBM-DTI-81 atlas; Fig. 3) were observed for the contrast intervention > control, with FA increasing more in the intervention group than in the control group. The contrast pre > post of the Control group showed significant voxels in the left optic radiation (FWE corrected *p*_*TFCE*_ = 0.09; JHU-ICBM-DTI-81 atlas; Supplementary File 4). No contrasts reached significance for the MD analysis. The RD analysis resulted in significant voxels for the ∆ (post-to-pre) datasets; in the Intervention < Control contrast, the inverse contrast to the effects in FA. The peak *p*_TFCE_ values of 0.07 was found in the body and splenium of the CC, both areas where the FA showed effects as well. There was a contralateral distribution of the measures with overlaps in the splenium (see the Z = 26 slice in Fig. 3). The contralateral distribution is especially apparent in the tapetum, in which RD under contrast intervention < control has a small effect size of 0.28.

 Regarding **neural correlates** of the observed improvements in cognitive performance, 12 WM tracts were identified that showed statistically significant weak to moderate correlations between changes in WM integrity and improvements in cognitive performance. These associations were mainly found around the thalamic radiation and CC and mostly moderate to weak strengths of associations (see Table 5). All remaining analyses can be found in the Supplementary File 5. Of note are the correlations of FA and RD of the ∆ FA in the CC– body and splenium– which showed the strongest associations with global cognitive performance, with correlation coefficients of 0.391 [0.132 to 0.650] and 0.372 [0.111 to 0.633], respectively.

**Table 5:** Neural correlates in white matter integrity to observed cognitive performance improvements

Region of Interest [normalized; % total intracranial volume]	Statistical Results		
	Correlation Coefficient (i.e., Pearson *r* (*r*) or Spearman rho (ρ)) [90% Confidence Interval]	*P* value	Sample size
*Neural Correlates to Global Cognitive Performance Adaptations*:			
∆ FA splenium of corpus callosum	*r* = 0.391 [0.132 to 0.650]	0.03	25
∆ FA posterior thalamic radiation - right	*r* = 0.372 [0.111 to 0.633]	0.04	25
∆ FA tapetum - right	*r* = 0.300 [0.030 to 0.570]	0.08	25
∆ FA body of corpus callosum	*r* = 0.272 [-0.001 to 0.543]	0.10	25
∆ RD splenium of corpus callosum	*r* = -0.491 [-0.730 to -0.252]	0.01	25
∆ RD body of corpus callosum	*r* = -0.378 [-0.639 to -0.117]	0.03	25
∆ RD posterior corona radiata - left	*r* = -0.348 [-0.612 to -0.084]	0.05	25
*Neural Correlates to Delayed Verbal Recall Adaptations*:			
∆ FA tapetum - right	ρ = 0.421 [0.101 to 0.664]	0.02	24^(1)^
∆ FA superior longitudinal fasciculus - right	ρ = 0.380 [0.111 to 0.637]	0.03	24^(1)^
∆ FA sagittal stratum - right	ρ = 0.311 [-0.035 to 0.618]	0.07	24^(1)^
∆ FA posterior thalamic radiation - right	ρ = 0.301 [-0.028 to 0.581]	0.08	24^(1)^
∆ FA superior corona radiata - right	ρ = 0.283 [-0.076 to 0.618]	0.09	24^(1)^

For readability, this table selectively illustrates the analyses between whiter matter integrity and previously reported cognitive improvements that returned a statistically significant correlation. All remaining analyses can be found in the supplementary file 5

(1) Missing data from one study participants for cognitive performance assessment because measurement was aborted due to emotional breakdown

Abbreviations: FA, fractional anisotropy; RD, radial diffusivity;

## Discussion

This is the first RCT exploring neural changes and their relationships to cognitive performance improvements following the addition of a co-designed, purpose-developed, and individually tailored exergame-based training to usual care in mNCD. The intervention was shown to be effective in improving global cognitive performance as well as immediate and delayed verbal recall [34]. It was delivered via the ‘Brain-IT’ training concept [34], which was iteratively co-designed, tested, and refined with patient and public involvement [26, 34].

### Principal findings

We observed moderate to large effects in favor of ‘Brain-IT’ training in GMVs of the hippocampus (total and right), thalamus (bilateral), left PFC, and left ACC, as well as WMVs of the total ACC. Changes in the hippocampus (total and right) and left thalamus were weakly positively correlated with improvements in verbal delayed recall. The identification of neural correlates to cognitive performance improvements aligns with the most recent systematic review on neurobiological effects in response to exergame-based training, however, implicated different ROIs. Specifically, Attoh-Mensah et al. (2025) [32] identified one published study that reported verbal memory changes to be related to changes in the dlPFC in mNCD [33] as opposed to the hippocampus and thalamus observed in this RCT. Given that only a single study with a small sample size (8 mNCD participants with complete MRI data) is available, we position our findings in relation to systematic reviews of the broader literature.

Attoh-Mensah et al. (2025) [32] also identified two RCTs on exergame-based training in healthy older adults, reporting inconsistent results on GMVs. For the broader literature on motor-cognitive training, Huang et al. (2022) [72] identified three studies including individuals with mNCD or Alzheimer’s dementia, reporting positive effects on GMV in the right hippocampus and bilateral ACC. Similarly, Yang et al. (2019) [73] reported on two RCTs with significant group differences– reduced GM atrophy in the frontal, parietal, entorhinal, and/or cingulate cortex in the intervention group– whereas two other RCTs reported no effects of motor-cognitive training on GMV. For the broader field of any type of physical exercise, Zhang et al. (2024) [74] reported no statistically significant effects on WMV or hippocampal volume in their meta-analysis.

Considering this state of evidence, our observations concur with the broader literature that some structural brain improvements can be achieved with motor-cognitive or exergame-based training, even in response to short-term interventions (in our case, 12 weeks). The specificity of the training may account for the heterogeneity in which brain regions improvements were measured. Specifically, given that most of our participants had Alzheimer’s etiology or were impaired mainly in the learning and memory domain of neurocognitive functioning, the ‘Brain-IT’ training concept tailored the multi-domain exercises accordingly. Our results suggest that this exercise specificity has induced the expected improvements, given that the intervention was effective for improving verbal recall [34], whereas its neural correlates are related mainly to brain areas implicated in these functions.

The hippocampus is well known to be involved in episodic and spatial memory [75], which aligns with our observation that the total GMV of the hippocampus may be a neural correlate of improvements in verbal delayed recall. However, some research indicates that the dominant– most often left– hippocampus is mostly involved in verbal memory while the non-dominant– often the right– hippocampus is involved in spatial and visual memory performance [76, 77]. Contrary to this prediction, we found that the ∆GMV of the right (not left) hippocampus may be a neural correlate of improvements in verbal delayed recall. Nevertheless, owing to the small sample size, weak correlations with CIs crossing zero, and lack of a visuospatial memory assessment, this observation must be interpreted with caution. Future research should confirm whether exergame-based training impacts hippocampal structure and whether, and to what extent, this explains changes in episodic and visuospatial memory.

Interestingly, we also found large effects on the GMV of the thalamus in favor of ‘Brain-IT’ training, which correlated with improvements in delayed recall. Potential explanations for this observation could be both the stimuli provided by the exergame-based training and HRV-BF training. Exergame-based training provides multisensory (i.e., visual, auditory, and tactile) stimuli and real-time feedback in multidimensional virtual environments. In this way, it challenges and trains (i) action-perception coupling via the coordination of perceptual inference about *“what is where”* from multi-sense-data, which requires planning and learning novel spatial motor movements, and (ii) the synergistic relationships between cardiovascular, muscular, perceptual motor, and cognitive subsystems [18], for both of which the thalamus is a key regulator [78–80]. An alternative explanation may be that the stimuli provided by HRV-BF training induced thalamic improvements, as it targets the central autonomous network, of which the thalamus is an integral part. This network is important for self-regulation and control of cognitive processes impaired in mNCD [70, 71]. The evidence suggests that HRV-BF training is effective for improving cognitive performance [81, 82] and emotional regulation [82–85], which may be explained by improvements in prefrontal structural integrity (particularly in the orbitofrontal cortex) [86]. This may help patients cope better with stressful environments, such as when faced with neuropsychological assessments. While these findings align with our results of moderate, albeit nonsignificant, effects on the left PFC, left ACC, and right thalamus, confirmatory studies are needed to elucidate the role of the central autonomous network as well as its substructures in behavioral adaptations to exergame-based training and/or HRV-BF training and to disentangle the potentially synergistic effects between the stimuli provided by these exercise modalities [34].

For WM integrity, we observed a potential protective effect (higher ΔFA and lower ΔRD values than those in the control group) in brain regions that were related to cognitive performance adaptations, mainly around the thalamic radiation and the CC. These findings cannot be directly compared to those in the literature in the field, as no previous exergame-based studies have analyzed changes in WM integrity in mNCD [32]. However, the broader literature on physical exercise reported significant increases in FA but no effects on RD or MD in older adults [74]. While this finding partially aligns with our observations, comparability is limited given that this review pooled the small number of studies that reported such outcomes in the meta-analyses, irrespective of any ROIs [74]. More systematic exploration of the effects of exergame-based training on WM structural integrity in older adults with and without mNCD is therefore needed. Nevertheless, our findings suggested that neural correlates of cognitive performance adaptations may be centered mainly around the thalamic radiation, which aligns with the findings on GMV and the CC.

The potential protective effect in the CC may be explained by the observation that patients with Alzheimer’s disease show extensive WM microstructural damage, structural disconnection, and topological abnormalities, mainly in the CC, fornix, and medial temporal lobe [4]. Such disruptions in the WM structure of the CC could in turn lead to hemispheric asymmetries, which are often observed in mNCD and have been proposed as neuroimaging markers for the prediction of the progression of mNCD to dementia, particularly in the entorhinal cortex [87]. Therefore, ‘Brain-IT’ training may have potential protective effects on brain regions that are well known to be affected by mNCD also at the WM structural level. However, hypothesis-driven studies to corroborate or refute these preliminary findings are warranted given the exploratory nature of our analyses.

### Strengths and limitations

This RCT had several important strengths and limitations, which were elaborated in detail in the publication of the parent RCT’s findings, including the rigorous development of the study’s intervention and generalizability of the findings in relation to the selection of study participants [34]. This study has three additional key limitations specific to neuroimaging analyses that are worth mentioning. First, and most importantly, the study was not powered for neuroimaging outcomes given that these were tertiary outcomes of the parent study [39]. While it represents the largest RCT on structural brain changes in response to exergame-based training in mNCD to date, the small sample size deems all analyses exploratory and requires adequately powered and hypothesis-driven studies to substantiate these initial findings. Second, in line with this exploratory approach, a second notable limitation is the choice of a higher than conventionally applied significance threshold (ɑ = 0.10) for the analyses of intervention-related effects. This decision is tempered by the study’s exploratory nature but necessitates cautious interpretation of the results focusing on effect estimates rather than relying solely on statistical significance, as detailed in the section *"General statistical procedures"*. Finally, while the inclusion of multiple disease etiologies of mNCD could be considered a strength in the parent study which was focused on generalizability and transferability to clinical practice in the context of an intervention that was tailored taking into account these different etiologies, it represents an inherent limitation of the analyses presented here. Specifically, due to the low sample size, it cannot be disentangled whether the training’s effect is a general effect shared by patients with different mNCD etiologies, or if it’s mainly coming from a specific group, like those with Alzheimer’s etiology.

### Implications for research

Hippocampal atrophy is a hallmark of Alzheimer’s disease with high prognostic value for disease progression [3–6]. This RCT’s results provide preliminary evidence for a potential association suggestive of a causal link between improvements at the neural (i.e., structural improvements in the hippocampus) and behavioral levels (i.e., improvements in verbal delayed recall [34]). While these observations may be interpreted as a first indication suggestive of a potential disease-modifying role of ‘Brain-IT’ training in mNCD, there are several shortcomings that need to be overcome in future studies to provide more robust evidence on the interventions effectiveness for secondary mNCD prevention.

First, we pooled data from both the intervention and control groups for our analyses on neural correlates. This decision was based on the observation of slight deterioration over time in both cognitive performance and/or structural brain outcomes in the control group, which was to be expected in a population with mNCD. A pooled analysis capturing the full range of individual changes– including improvements (mainly expected and observed in the intervention group) and slight declines (mainly expected and observed in the control group)– was therefore conducted. This approach benefited statistical power in this exploratory context, whereas single-group analysis would have suffered from too small sample sizes, particularly for the non-parametric analyses. Therefore, future research should aim for sufficiently large sample sizes to enable more robust, group-specific analyses and to disentangle whether the relationships between changes in cognitive performance and structural or functional brain changes differ between exercise and control interventions.

Second, our results provide some indication that the neural correlates of cognitive performance changes might be more closely related to changes in white matter, as compared to gray matter (see Tables 3 and 5). While there is emerging evidence suggesting that physical exercise can modulate human adult hippocampal neurogenesis and thereby impact on hippocampal-related cognition [88], this observation potentially indicates that the structural brain adaptations might be more closely linked to synaptic plasticity as compared to neurogenesis. In fact, synaptic plasticity is considered the direct target of exercise in the brain [9]. However, it has been suggested that much broader evaluations might be required to fully capture and understand the complex mechanisms, which might include but are not limited to neurotrophic factors, mitochondrial biogenesis, kinetics, and autophagy, or specific evaluations of the muscle-brain axis, the gut-brain axis and the liver-brain axis [9]. Therefore, further research should follow-up with hypothesis-driven studies to shed more light into the potential neurobiological mechanisms of action of exergame-based training on brain structure.

Third, while the observed short-term effects are important first indications for the interventions’ effectiveness for secondary prevention, no RCTs have investigated whether (exergame-based) physical or multidomain training can prevent or delay the onset of dementia in mNCD [16]. Therefore, future RCTs should prioritize providing empirical evidence on its potential long-term disease-modifying effects by investigating disease progression as the primary outcome [16].

Finally, more systematic evaluations of potential brain changes induced by exergame-based training, expanding to functional brain changes as well as appropriately powered RCTs with prespecified hypotheses for specific ROIs, are needed [32]. Once the neurobiological effects of exergame-based training and its moderators are better understood, this information can be fed back into optimizing the design of serious exergames and exergame-based training concepts [32].

## Conclusion

This is the first RCT showing that purpose-developed and individually tailored exergame-based training may positively impact brain structures affected in mNCD. Specifically, we observed positive structural changes in the hippocampus and subregions of the central autonomous network, including the thalamus and ACC. This RCT’s results also provide preliminary evidence for a potential association suggestive of a causal link between improvements at the neural and behavioral levels. Specifically, structural improvements in the hippocampus correlated with improvements in verbal delayed recall. Given that hippocampal atrophy is a hallmark of Alzheimer’s disease with high prognostic value for disease progression [3–6], this may be a first indication of a potential disease-modifying role of ‘Brain-IT’ training in mNCD. However, long-term studies on the potential disease-modifying effects of (technology-enhanced) physical and/or motor-cognitive training by investigating disease progression as the primary outcome are needed to provide conclusive evidence on this matter [16]. Given the exploratory nature of this initial study, adequately powered and hypothesis-driven studies should build on these findings by performing more systematic evaluations of potential brain changes induced by exergame-based training and expanding to include functional brain changes. Once the neurobiological effects of exergame-based training are better understood, this information can be fed back into optimizing the design of serious exergames and exergame-based training concepts [32]. These efforts should aim to provide optimal stimuli to promote neuroplastic changes and influence the pathological mechanisms of mNCD, thereby ultimately maximizing the effectiveness of exergame-based training in the secondary prevention of mNCD.

## Supplementary Information

Below is the link to the electronic supplementary material.

Supplementary Material 1

## Data Availability

The study protocol for this RCT was published previously and is available via open access [39]. The datasets that were generated and analyzed during the current study are available in the Zenodo repository at https://doi.org/10.5281/zenodo.10695988 for all data on the study populations as well as cognitive performance outcomes. Data from MRI scans are not publicly available owing to the sensitive nature of neuroimaging data but are available from the corresponding author upon reasonable request.

## Abbreviations

ACC: Anterior cingulate cortex
ANCOVA: Analysis of covariance
CC: Corpus callosum
Cis: Confidence intervals
CONSORT: Consolidated Standards of Reporting Trials
dlPFC: Dorsolateral prefrontal cortex
DSM-5: Diagnostic and Statistical Manual of Mental Disorders 5th Edition
DTI: Diffusion tensor imaging
FA: Fractional anisotropy
FEW: Family-wise error
GMC: Gray matter volume
ICD-XI: International Classification of Diseases 11th Revision
MD: Mean diffusivity
MIDE: Multidisciplinary Iterative Design of Exergames
mNCD: Mild neurocognitive disorder
MRI: Magnetic resonance imaging
η^2^_p_: Partial eta squared
PFC: Prefrontal cortex
r: Pearson r
RD: Radial diffusivity
ROI: Regions of interest
T_1_w: T1 weighted
TBSS: Tract-based spatial statistical
TFCE: Threshold-free cluster enhancement
WL: Web link
WM: White matter
WMV: Whiter matter volume
ρ: Spearman rho
