# Supplementary material for: Structural brain improvements following individually tailored serious exergame-based training in mild neurocognitive disorders: exploratory randomized controlled trial
Source: Alzheimers Res Ther. 2025 Sep 8;17:190. doi: 10.1186/s13195-025-01835-2 (PMC12416070; doi:10.1186/s13195-025-01835-2)
Supplement: Supplementary file 1 — Supplementary Material 1 [file 13195_2025_1835_MOESM1_ESM.pdf]

Supplementary Files to Publication:

Structural Brain Improvements Following Individually Tailored Serious Exergame-based Training in Mild Neurocognitive Disorders: A Randomized Controlled Trial

Patrick Manser[1, 2]*, Michael Rosio[3], André Schmidt[4], Lars Michels[3, 5], and Eling D. de Bruin[1, 2, 6]

[1] Motor Control and Learning Group, Institute of Human Movement Sciences and Sport, Department of Health Sciences and Technology, ETH Zurich, Zurich, Switzerland

[2] Division of Physiotherapy, Department of Neurobiology, Care Sciences, and Society, Karolinska Institute, Huddinge, Sweden

[3] Clinical Neuroscience Center, Department of Neuroradiology, University Hospital Zurich, Zurich, Switzerland

[4] Department of Psychiatry, University of Basel, Basel, Switzerland

[5] Neuroscience Center Zurich, University of Zurich and ETH Zurich, Zurich, Switzerland.

[6] Department of Health, OST - Eastern Swiss University of Applied Sciences, St. Gallen, Switzerland

16-digit ORCID of the author(s):

Patrick Manser	ORCID: 0000-0003-3300-6524
Michael Rosio	ORCID: 0009-0001-2860-8435
André Schmidt	ORCID: 0000-0001-6055-8397
Lars Michels	ORCID: 0000-0003-3750-1100
Eling D. de Bruin	ORCID: 0000-0002-6542-7385

*Correspondence:

Patrick Manser | Dr. sc. ETH Zurich
Department of Neurobiology, Care Sciences and Society | Karolinska Institutet
Alfred Nobels Allé 23 | 14183 Huddinge
patrick.manser@ki.se | https://www.patrick-manser.com/

Supplementary File 1 - CONSORT checklist

Table 1: 2017 CONSORT Checklist of Information to Include When Reporting Randomized Trials Assessing nonpharmacologic treatments [1]

Section/Topic:	Item No:	Checklist item:	Reported in section(s):
**TITLE AND ABSTRACT:**			
	1a	Identification as a randomized trial in the title.	'Title'
	1b	Structured summary of trial design, methods, results, and conclusions (for specific guidance see CONSORT for abstracts).	'Abstract'.
**INTRODUCTION:**			
Background and objectives:	2a	Scientific background and explanation of rationale.	'Introduction' and 'Materials and Methods - Prior Work'.
	2b	Specific objectives or hypotheses.	'Materials and Methods - Objectives and Hypotheses'; more details in the published study protocol [2].
**METHODS:**			
Trial design:	3a	Description of trial design (such as parallel, factorial) including allocation ratio. When applicable, how care providers were allocated to each trial group.	'Materials and Methods - Overview of the Trial Design, Participants, and Interventions'; more details in the published study protocol [2] and previous publication of the parent RCT's findings [3].
	3b	Important changes to methods after trial commencement (such as eligibility criteria), with reasons.	'Materials and Methods – Important modifications to the study protocol'.
Participants:	4a	Eligibility criteria for participants. When applicable, eligibility criteria for centers and for care providers.	'Materials and Methods - Overview of the Trial Design, Participants, and Interventions'; more details in the published study protocol [2] and previous publication of the parent RCT's findings [3].
	4b	Settings and locations where the data were collected.	'Materials and Methods - Overview of the Trial Design, Participants, and Interventions'; more details in the published study protocol [2] and previous publication of the parent RCT's findings [3].
Interventions:	5	The interventions for each group with sufficient details to allow replication, including how and when they were actually administered. Precise details of both the experimental treatment and comparator.	'Materials and Methods - Overview of the Trial Design, Participants, and Interventions' and 'Materials and Methods - Exergame Training with Biofeedback Breathing - The 'Brain-IT' Training Concept'; more details in the published study protocol [2] and previous publication of the parent RCT's findings [3].
	5a	Description of the different components of the interventions and, when applicable, description of the procedure for tailoring the interventions to individual participants.	'Materials and Methods - Overview of the Trial Design, Participants, and Interventions' and 'Materials and Methods - Exergame Training with Biofeedback Breathing - The 'Brain-IT' Training Concept'; more details in the published study protocol [2] and previous publication of the parent RCT's findings [3].
	5b	Details of whether and how the interventions were standardized.	'Materials and Methods - Overview of the Trial Design, Participants, and Interventions' and 'Materials and Methods - Exergame Training with Biofeedback Breathing - The 'Brain-IT' Training Concept'; more details in

			the published study protocol [2] and previous publication of the parent RCT's findings [3].
	5c	Details of whether and how adherence of care providers to the protocol was assessed or enhanced.	N/A
	5d	Details of whether and how adherence of participants to interventions was assessed or enhanced.	Previous publication of the parent RCT's findings [3].
Outcomes:	6a	Completely defined pre-specified primary and secondary outcome measures, including how and when they were assessed.	'Materials and Methods - Outcomes and Analyses'; more details in the published study protocol [2].
	6b	Any changes to trial outcomes after the trial commenced, with reasons.	N/A
Sample size:	7a	How sample size was determined. When applicable, details of whether and how the clustering by care providers or centers was addressed.	'Materials and Methods - Sample Size'; more details in the published study protocol [2].
	7b	When applicable, explanation of any interim analyses and stopping guidelines.	N/A (see 'Materials and Methods – Outcomes and Analyses')
Randomization:			
Sequence generation:	8a	Method used to generate the random allocation sequence.	'Materials and Methods - Randomization'; more details in the published study protocol [2].
	8b	Type of randomization; details of any restriction (such as blocking and block size).	'Materials and Methods - Randomization'; more details in the published study protocol [2].
Allocation concealment mechanism:	9	Mechanism used to implement the random allocation sequence (such as sequentially numbered containers), describing any steps taken to conceal the sequence until interventions were assigned.	'Materials and Methods - Randomization'; more details in the published study protocol [2].
Implementation:	10	Who generated the random allocation sequence, who enrolled participants, and who assigned participants to Interventions.	'Materials and Methods - Randomization'; more details in the published study protocol [2].
Blinding:	11a	If done, who was blinded after assignment to interventions (e.g., participants, care providers, those administering co-interventions, those assessing outcomes) and how.	'Materials and Methods - Blinding'; more details in the published study protocol [2].
	11b	If relevant, description of the similarity of interventions.	N/A
	11c	If blinding was not possible, description of any attempts to limit bias	N/A
Statistical methods:	12a	Statistical methods used to compare groups for primary and secondary outcomes. When applicable, details of whether and how the clustering by care providers or centers was addressed.	'Materials and Methods – Outcomes and Analyses'
	12b	Methods for additional analyses, such as subgroup analyses and adjusted analyses	N/A (see 'Materials and Methods – Outcomes and Analyses')
**RESULTS:**			
Participant flow (a diagram is strongly recommended):	13a	For each group, the numbers of participants who were randomly assigned, received intended treatment, and were analyzed for the primary outcome. The number of care providers or centers performing the intervention in each group and the number of patients treated by each care provider or in each center.	'Results'; more details in previous publication of the parent RCT's findings [3]
	13b	For each group, losses and exclusions after randomization, together with reasons.	Previous publication of the parent RCT's findings [3].

	13c	For each group, the delay between randomization and the initiation of the intervention.	Previous publication of the parent RCT's findings [3].
	13d	Details of the experimental treatment and comparator as they were implemented.	Previous publication of the parent RCT's findings [3].
Recruitment:	14a	Dates defining the periods of recruitment and follow-up.	Previous publication of the parent RCT's findings [3].
	14b	Why the trial ended or was stopped.	Previous publication of the parent RCT's findings [3].
Baseline data:	15	A table showing baseline demographic and clinical characteristics for each group. When applicable, a description of care providers (case volume, qualification, expertise, etc.) and centers (volume) in each group.	'Results - Baseline Data'
Numbers analyzed:	16	For each group, number of participants (denominator) included in each analysis and whether the analysis was by original assigned groups.	'Results' and Tables 1 - 5
Outcomes and estimation:	17a	For each primary and secondary outcome, results for each group, and the estimated effect size and its precision (such as 95% confidence interval).	'Results', Tables 1 - 5, and Supplementary Files
	17b	For binary outcomes, presentation of both absolute and relative effect sizes is recommended	N/A
Ancillary analyses:	18	Results of any other analyses performed, including subgroup analyses and adjusted analyses, distinguishing pre-specified from exploratory	N/A (see 'Materials and Methods – Outcomes and Analyses')
Harms:	19	All important harms or unintended effects in each group (for specific guidance see CONSORT for harms)	Previous publication of the parent RCT's findings [3].
**DISCUSSION:**			
Limitations:	20	Trial limitations, addressing sources of potential bias, imprecision, and, if relevant, multiplicity of analyses. In addition, take into account the choice of the comparator, lack of or partial blinding, and unequal expertise of care providers or centers in each group.	'Discussion - Strength and Limitations' and previous publication of the parent RCT's findings [3].
Generalizability:	21	Generalizability (external validity) of the trial findings according to the intervention, comparators, patients, and care providers and centers involved in the trial.	'Discussion - Principal Findings' and 'Discussion - Strength and Limitations'
Interpretation:	22	Interpretation consistent with results, balancing benefits - and harms, and considering other relevant evidence.	'Discussion - Principal Findings' and 'Discussion - Implications for Research'
**OTHER INFORMATION:**			
Registration:	23	Registration number and name of trial registry.	'Abstract' and 'Materials and Methods - Protocol, Registration, and Reporting'
Protocol:	24	Where the full trial protocol can be accessed, if available.	'Materials and Methods - Protocol, Registration, and Reporting'
Funding:	25	Sources of funding and other support (such as supply of drugs), role of funders.	'Funding Sources'

Supplementary File 2 - Results of Scan Quality Checks based on Segmentation and Sample Homogeneity Tools from the Computational Anatomy Toolbox for SPM

T1-weighted MPRAGE scan quality was assessed in the Computational Anatomy Toolbox for SPM (CAT12). Specifically, we used the CAT12 segmentation and sample homogeneity toolboxes, providing easily interpretable quality measures at the participant and group level. The weighted overall image quality (IQR) and the quartic mean Z-score are the two key indicators of image quality. IQR combines noise and spatial resolution measurements before pre-processing, while the mean quartic Z-score assesses the homogeneity of data after pre-processing, with deviations increasing variance and reducing statistical power. The product of IQR and quartic mean Z-scores combines these quality measures, with a low number indicating high quality.

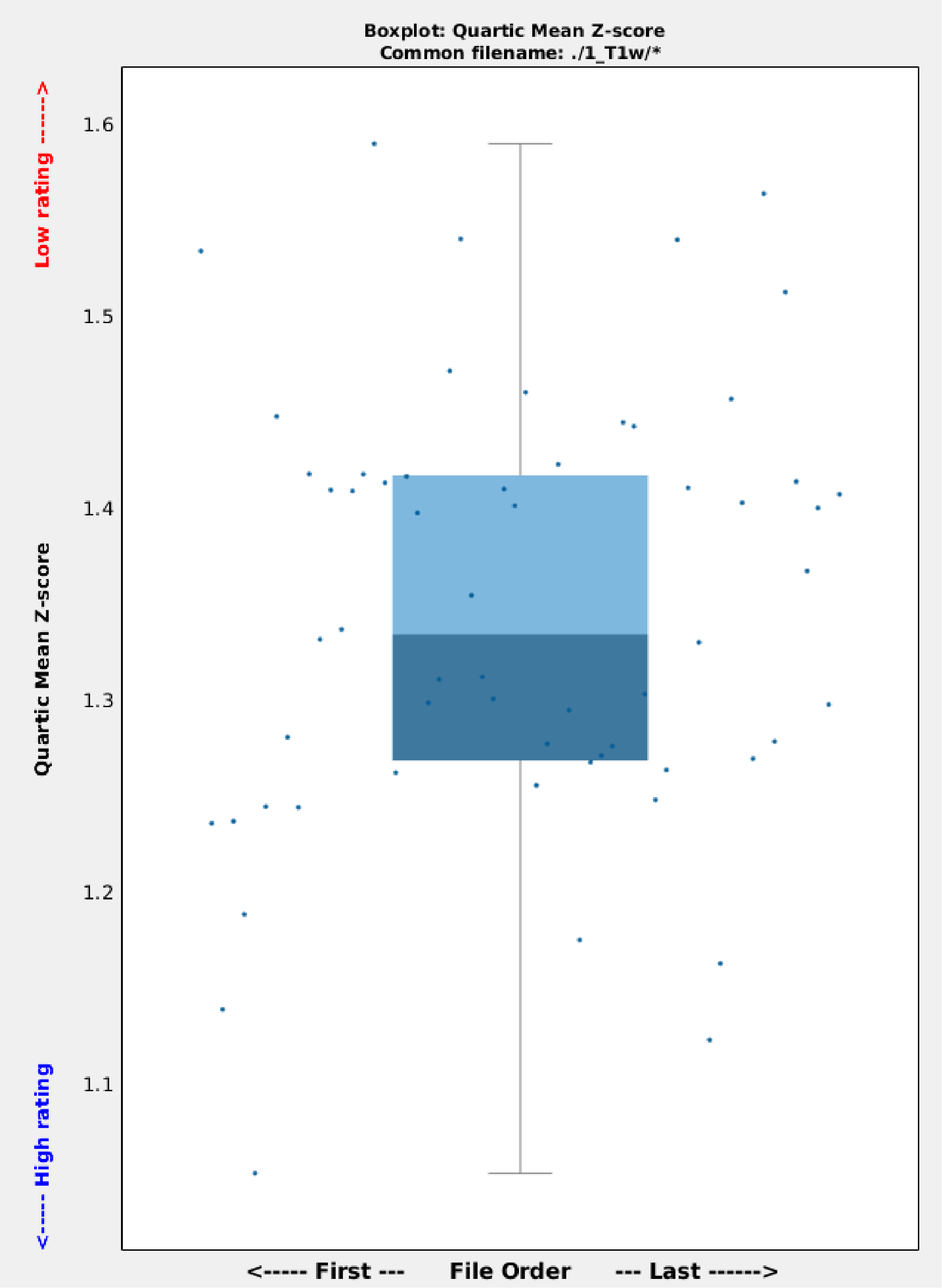

CAT12 output. Boxplot showing the weighted IQR by mean absolute z-score scaled by a factor of 4 (i.e. quartic) to emphasize outliers. As the plot shows, all participants in this distribution lie in the good to optimal range of the quality measure. Of note, even when outliers are detected, these are not necessarily data to discard on an absolute basis, as the score is calculated relative to the specific sample being analyzed.

Supplementary File 3 - Neural correlates in gray and white matter volume changes to observed cognitive performance improvements

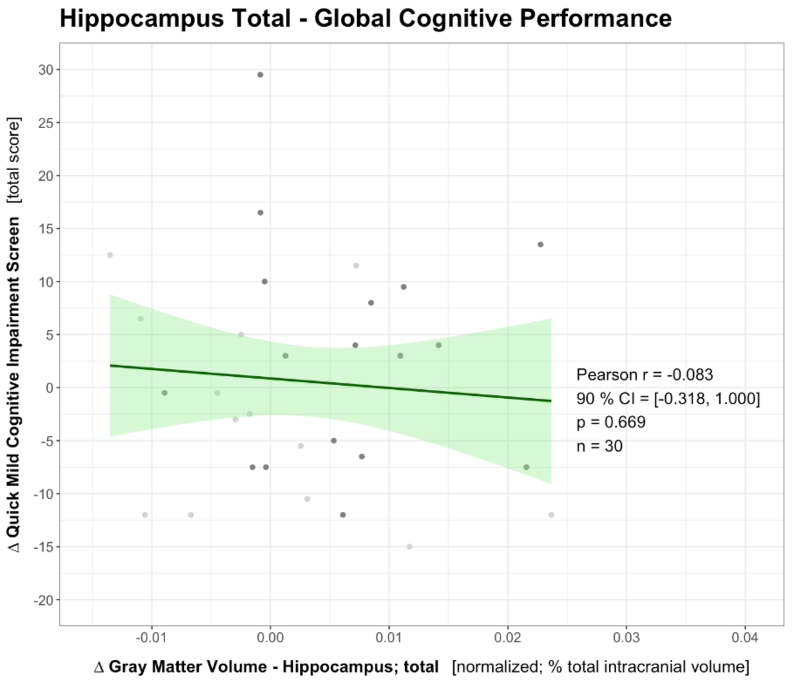

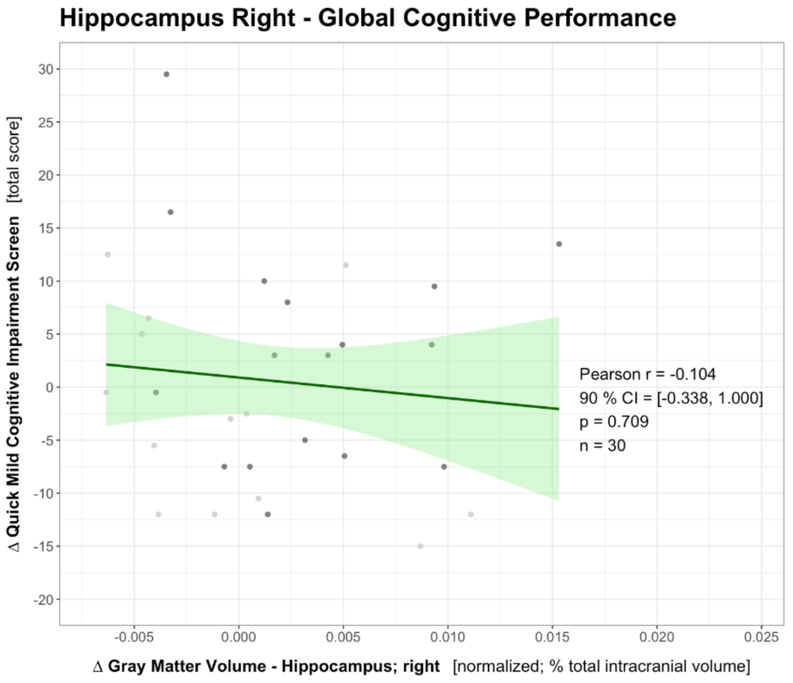

Legend: light gray = Control Group
dark gray = Intervention Group

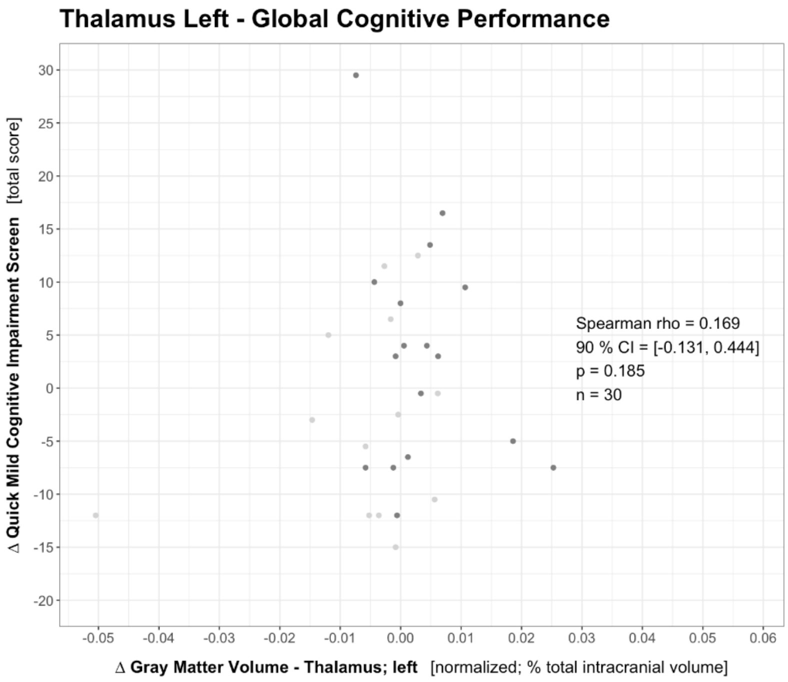

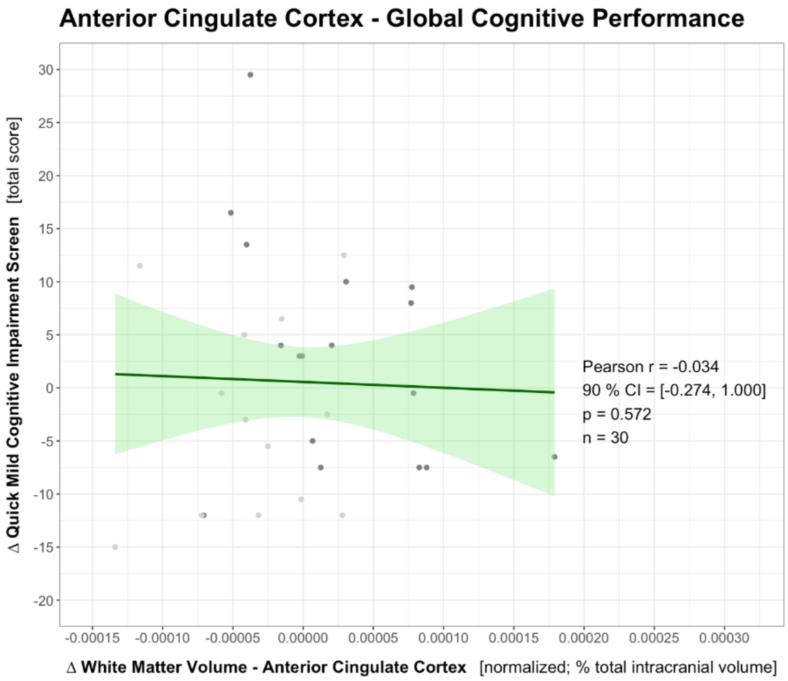

Legend: light gray = Control Group
dark gray = Intervention Group

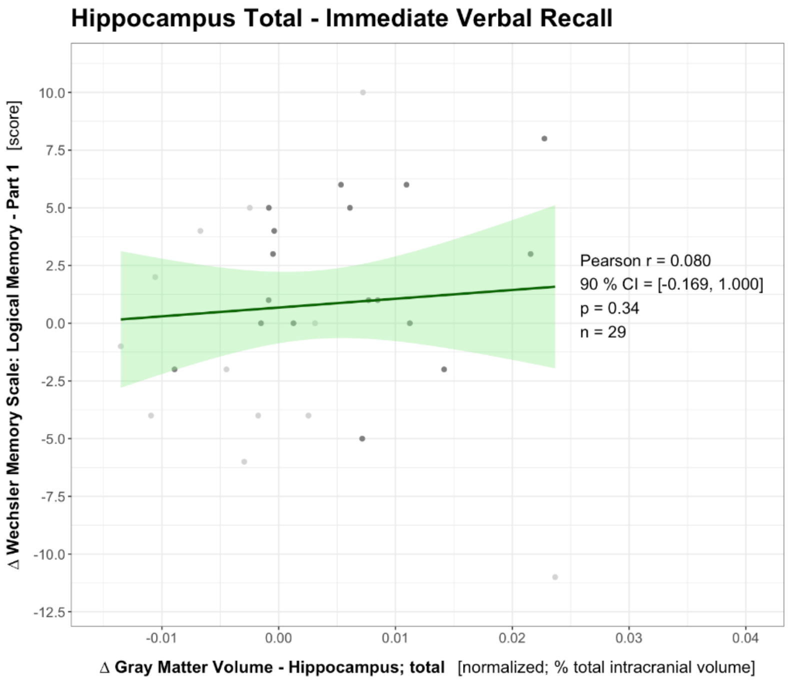

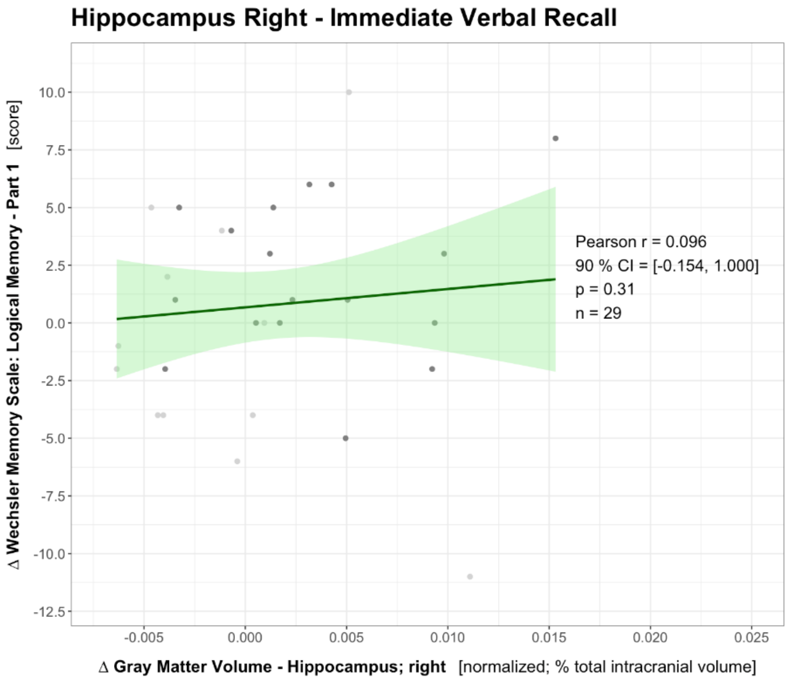

<u>Legend:</u> light gray = Control Group
dark gray = Intervention Group

Thalamus Left - Immediate Verbal Recall

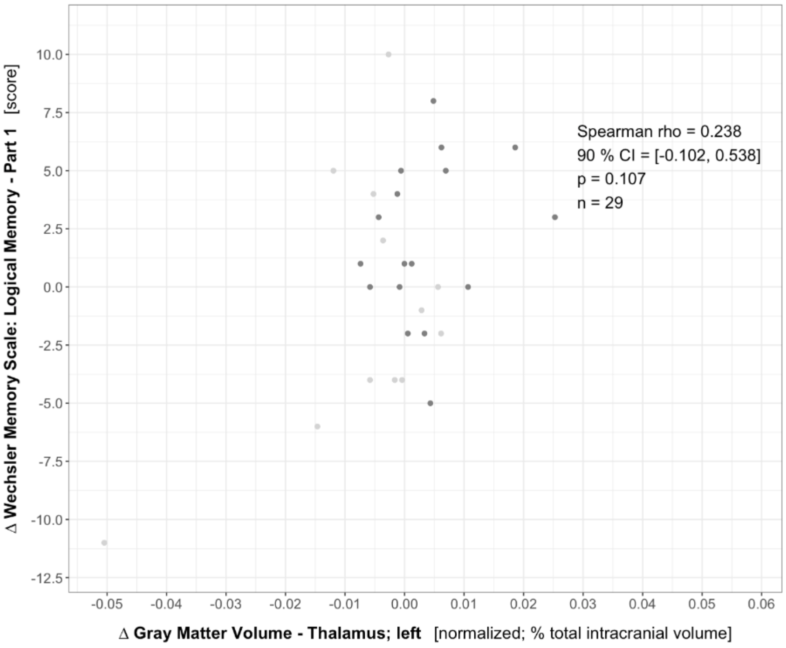

Anterior Cingulate Cortex - Immediate Verbal Recall

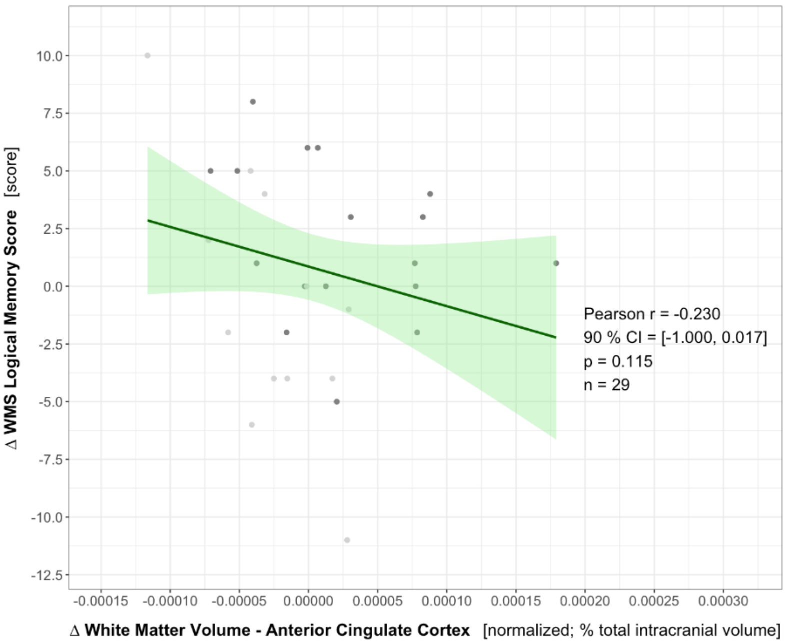

Legend: light gray = Control Group
dark gray = Intervention Group

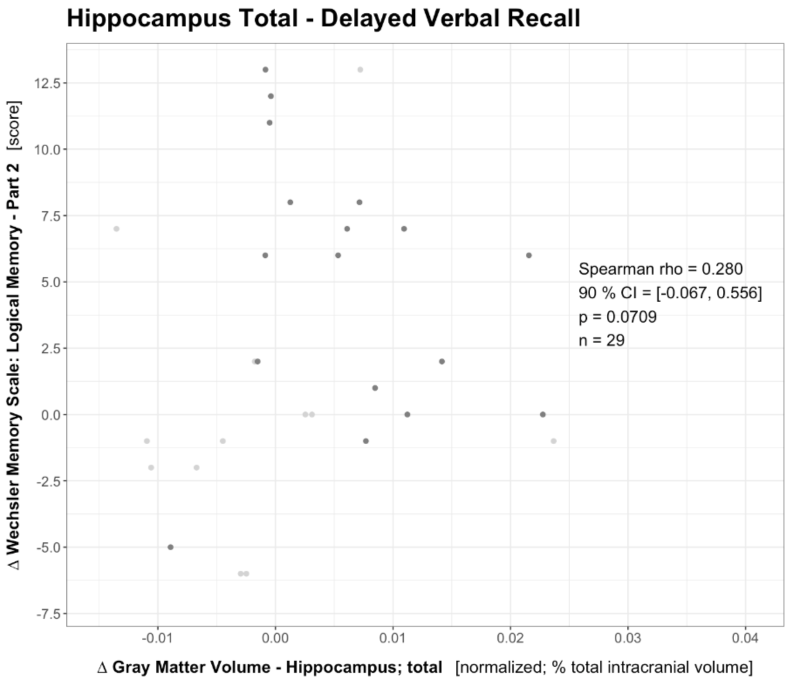

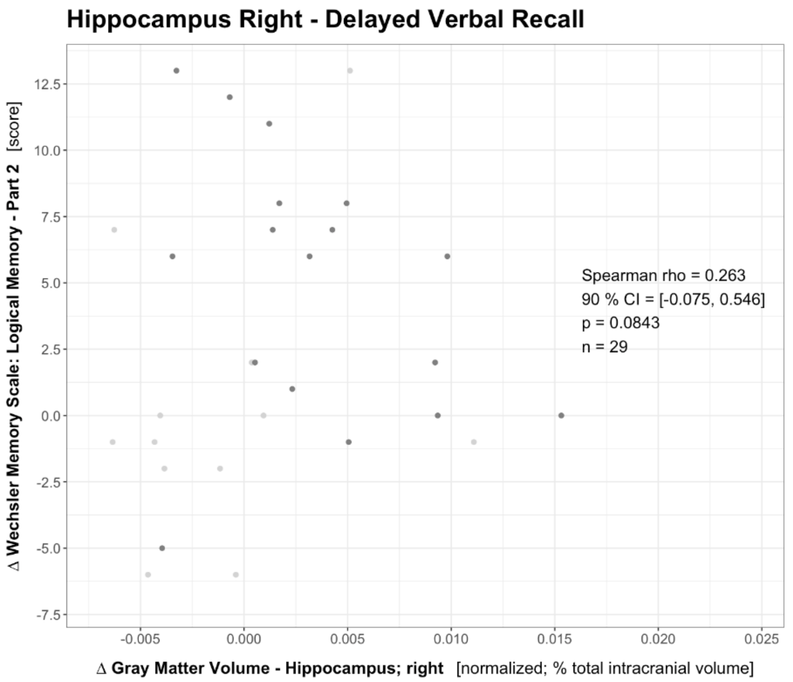

Legend: light gray = Control Group
dark gray = Intervention Group

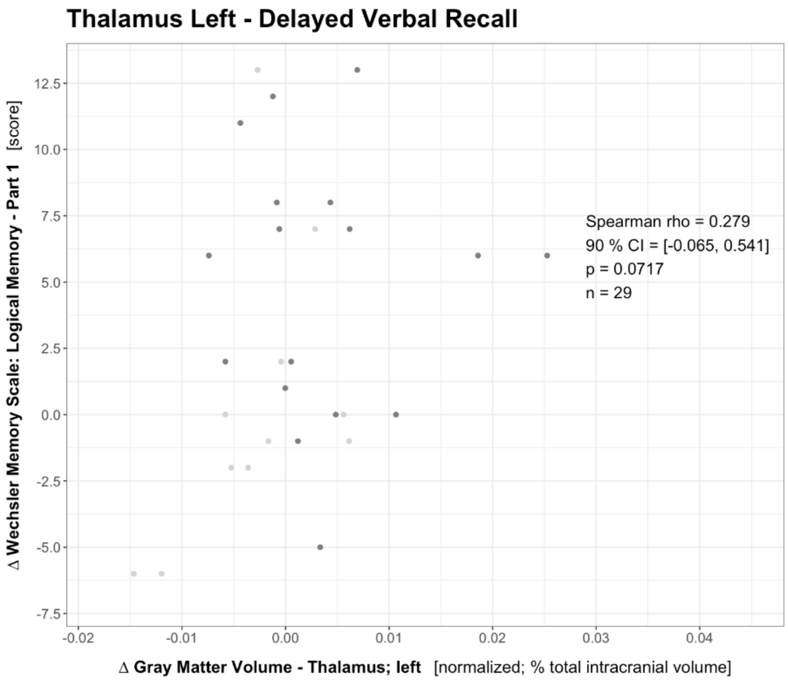

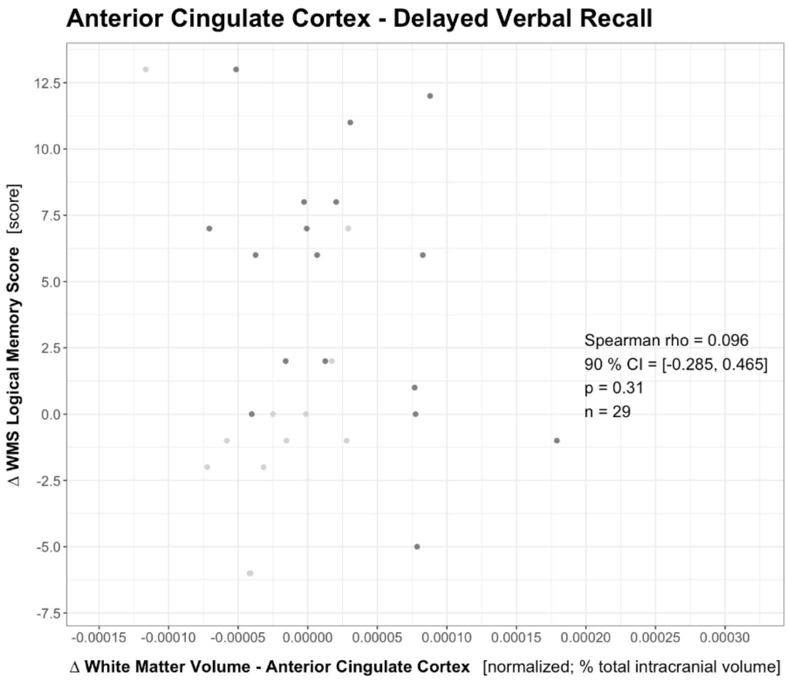

Legend: light gray = Control Group
dark gray = Intervention Group

Supplementary File 4 - Supplementary Figure on the Pre > Post Contrast on the Optic Radiation in the White Matter Integrity Analysis

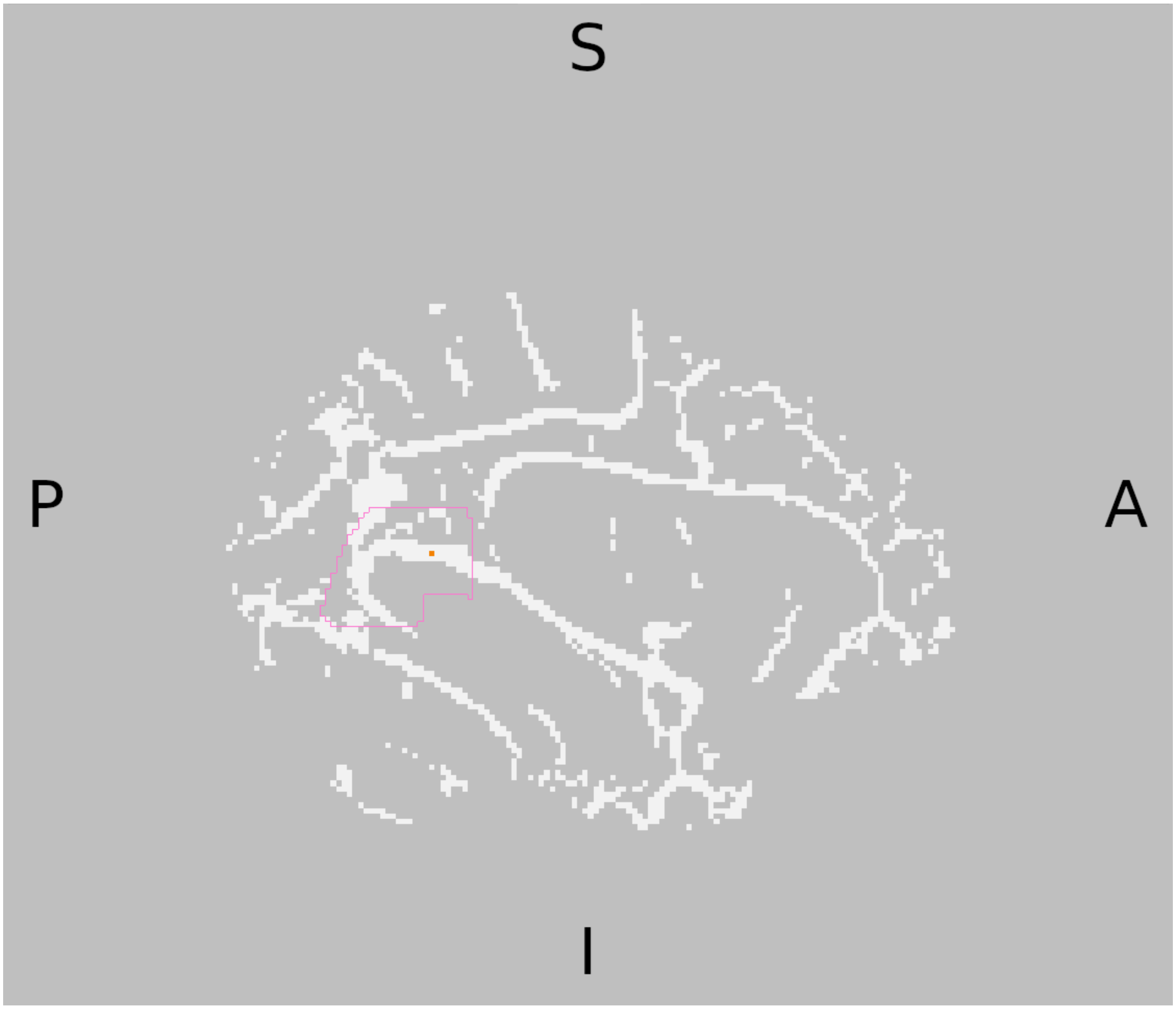

FA image of the contrast pre > post of the Control group showing significant voxels in the left optic radiation (FWE corrected p = 0.09). The frame shown in the picture represents the "Left Posterior thalamic radiation (includes optic radiation)" of the JHU-ICBM-DTI-81 atlas.

Supplementary File 5 - Full Results on Neural Correlates to Observed Improvements in Cognitive Performance from White Matter Integrity Analyses

WMS LM1 total score
Total

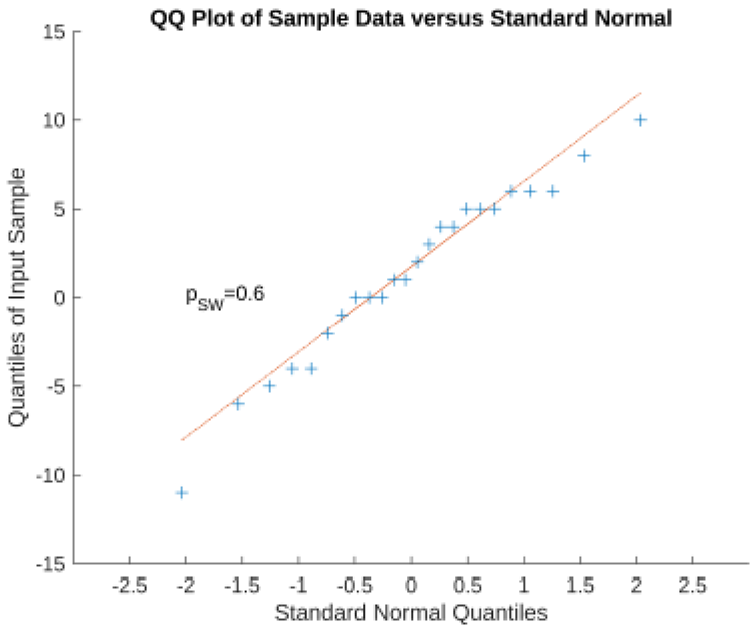

WMS LM2 total score
Total

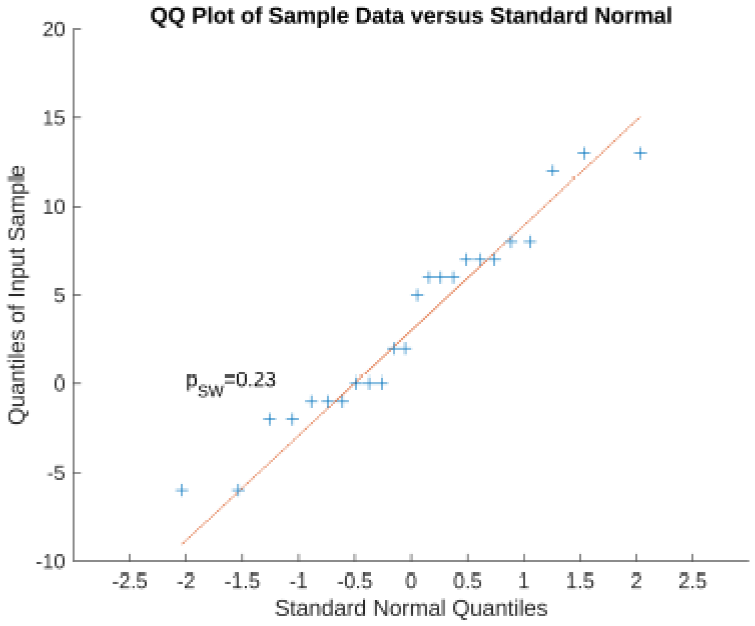

Qmci score tot
Total

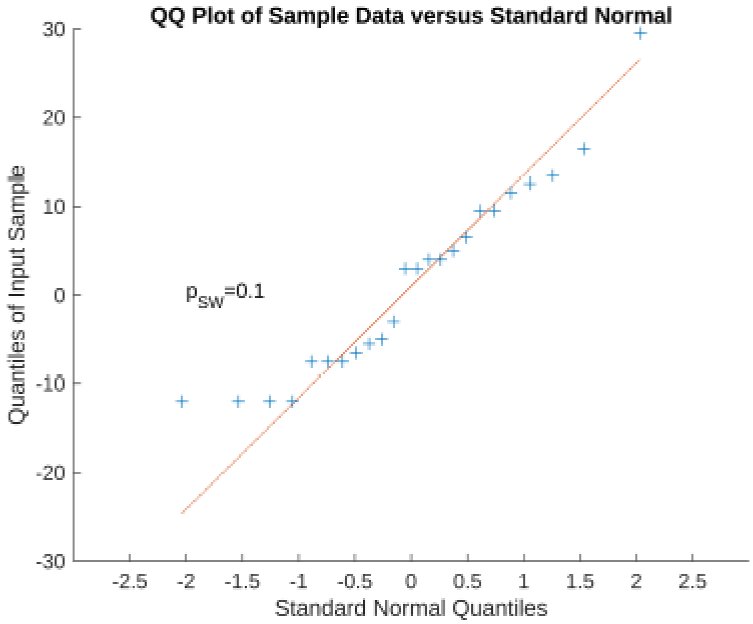


```
Body of corpus callosum
FA~WMS LM1 total score
```

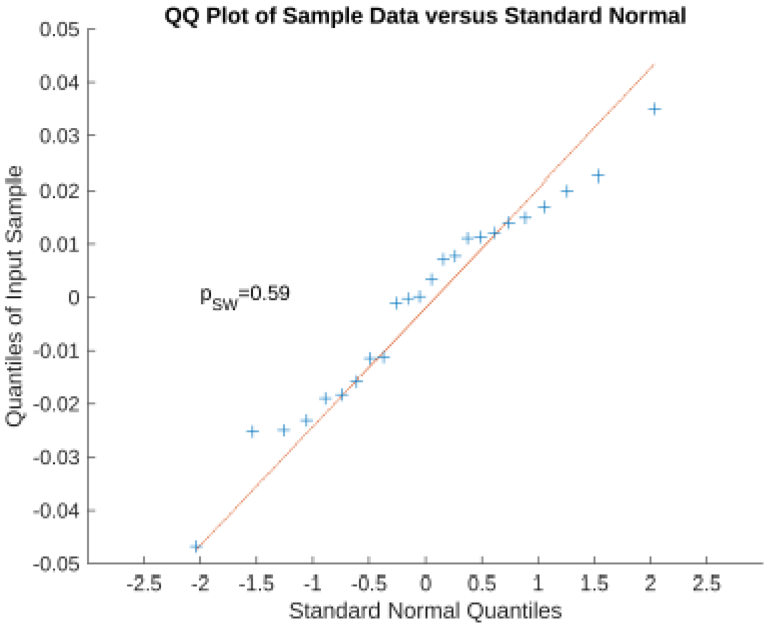


```
Pearson R
 r=-0.18, p=0.8
Splenium of corpus callosum
FA~WMS LM1 total score
```

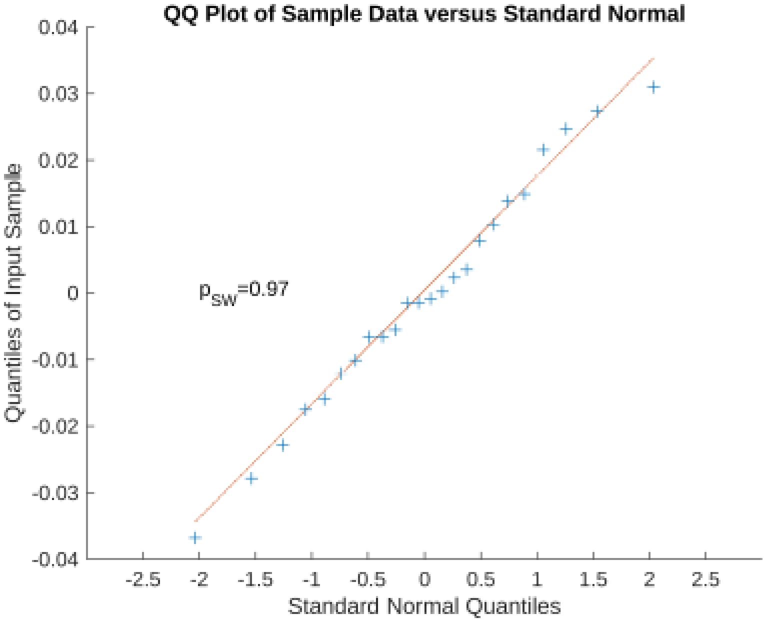

Pearson R
r=-0.057, p=0.6
Retrolenticular part of internal capsule R
FA~WMS LM1 total score

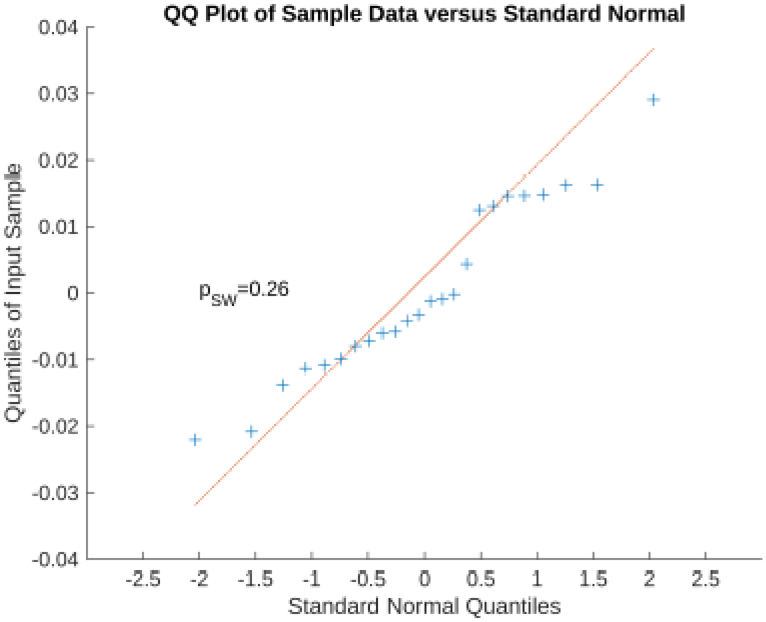

Pearson R
r=0.18, p=0.21
Superior corona radiata R
FA~WMS LM1 total score

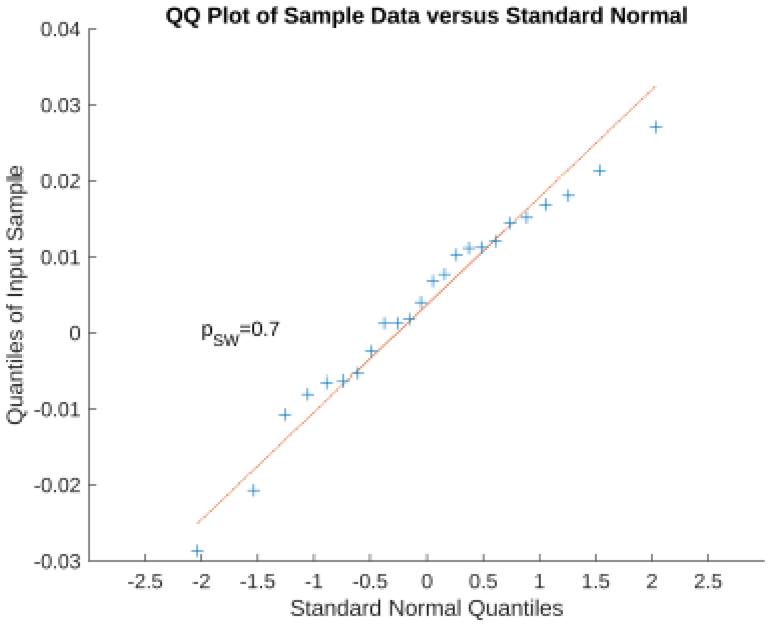

Pearson R
r=-0.085, p=0.65
Posterior corona radiata R
FA~WMS LM1 total score

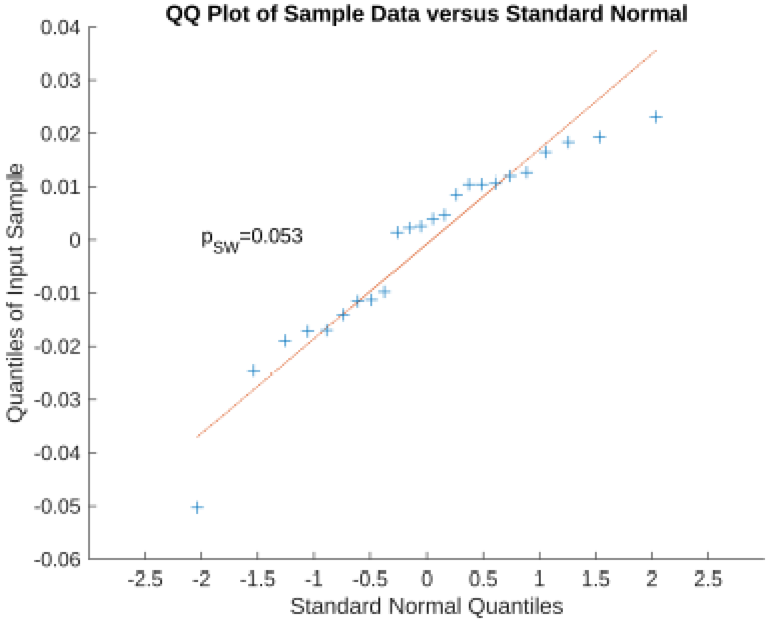

Pearson R
r=-0.2, p=0.83
Posterior thalamic radiation R
FA~WMS LM1 total score

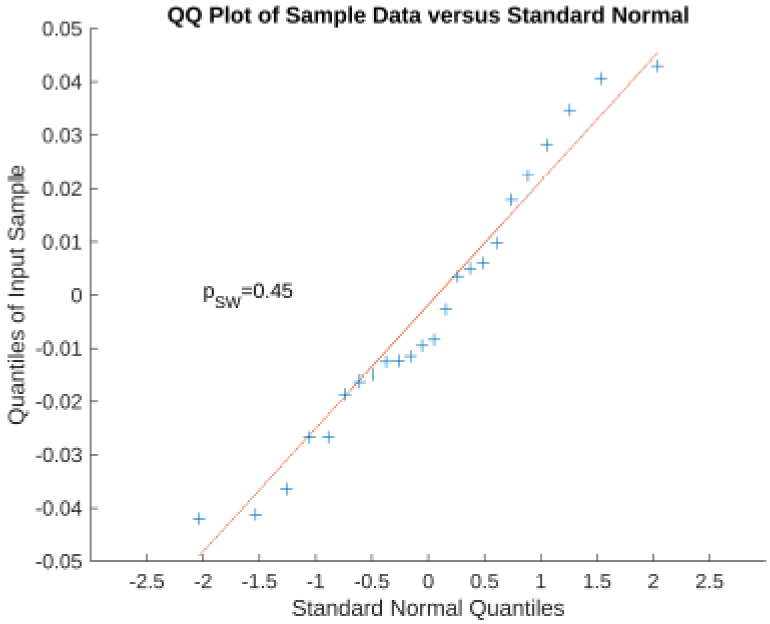

Pearson R
r=-0.07, p=0.63
Sagittal stratum R
FA~WMS LM1 total score

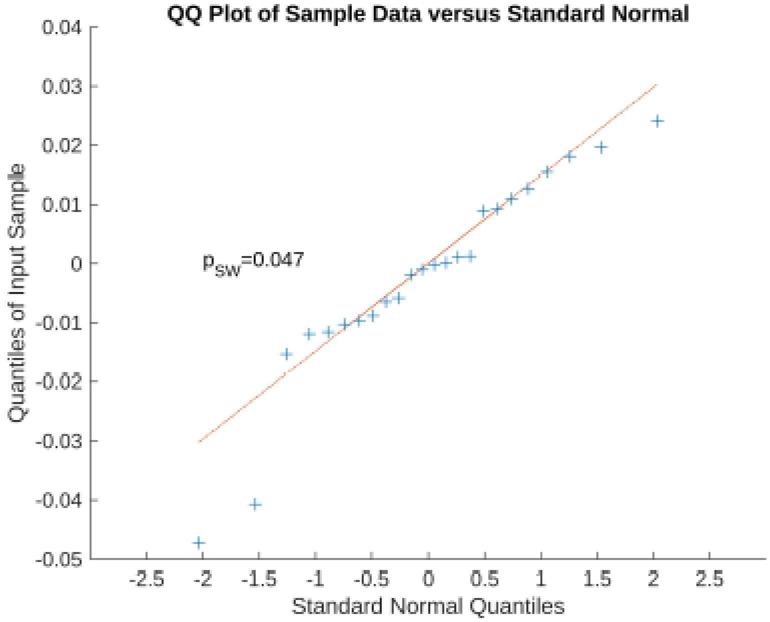

Spearman's Rho
ρ=-0.055, p=0.6
Superior longitudinal fasciculus R
FA~WMS LM1 total score

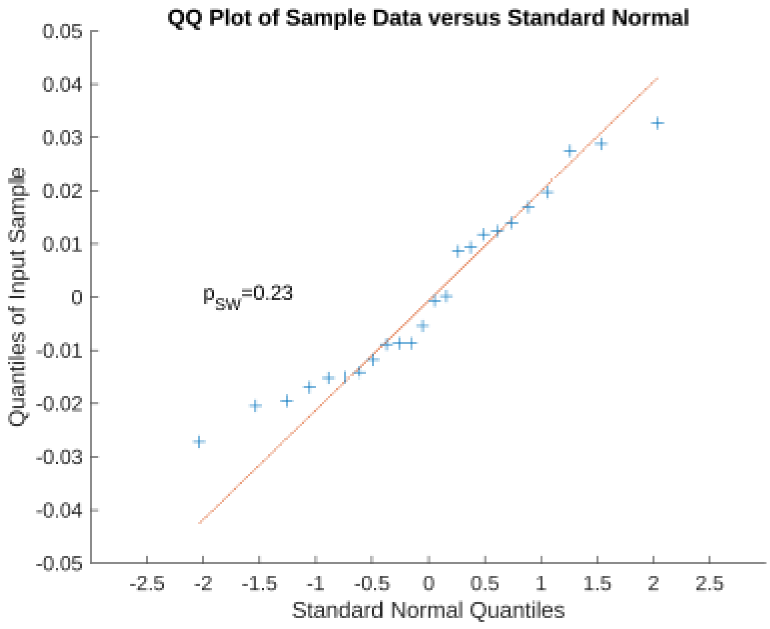


```
Pearson R
 r=0.049, p=0.41
Tapetum R
FA~WMS LM1 total score
```

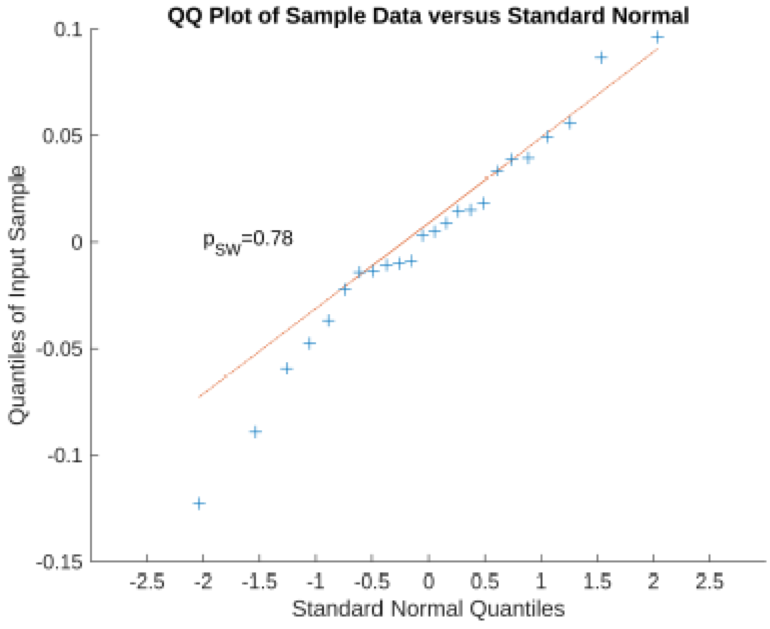


```
Pearson R
 r=-0.18, p=0.8
Body of corpus callosum
FA~WMS LM2 total score
```

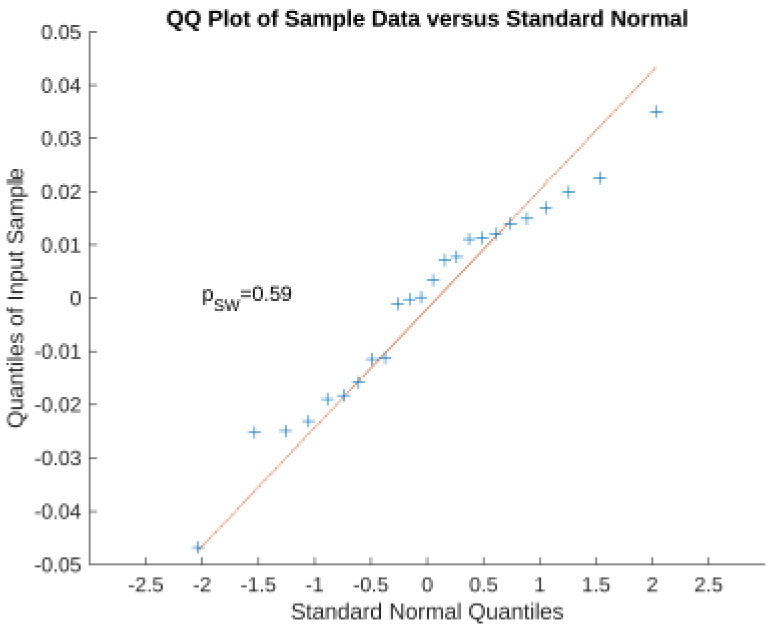

Spearman's Rho
ρ=0.062, p=0.39
Splenium of corpus callosum
FA~WMS LM2 total score

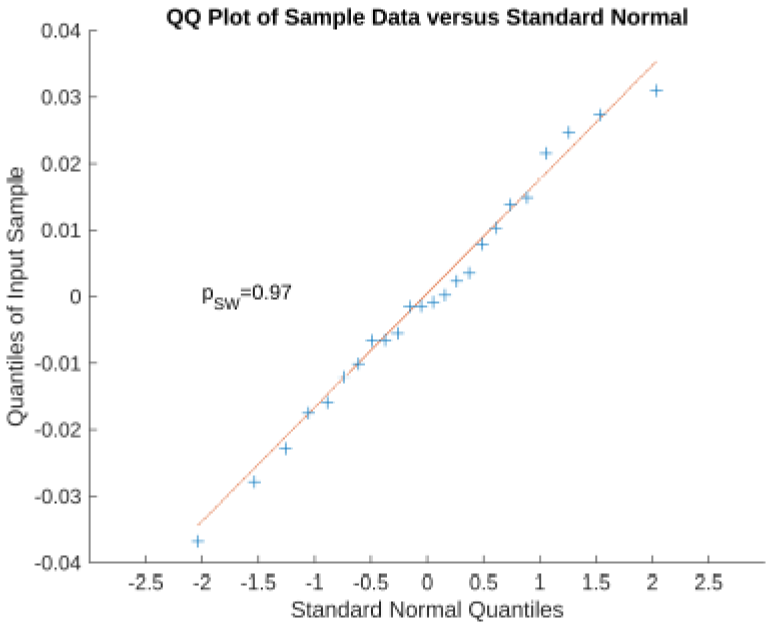

Spearman's Rho
ρ=0.19, p=0.19
Retrolenticular part of internal capsule R
FA~WMS LM2 total score

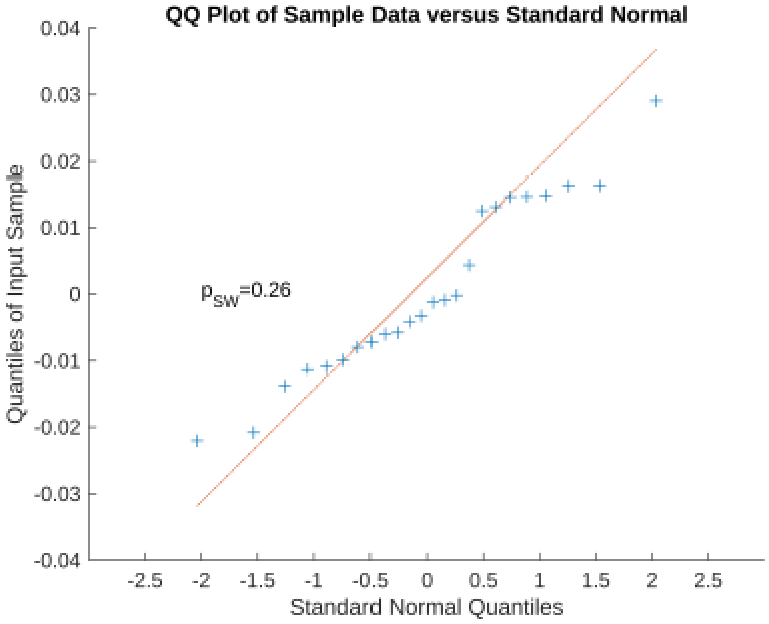

Spearman's Rho
ρ=0.057, p=0.4
Superior corona radiata R
FA~WMS LM2 total score

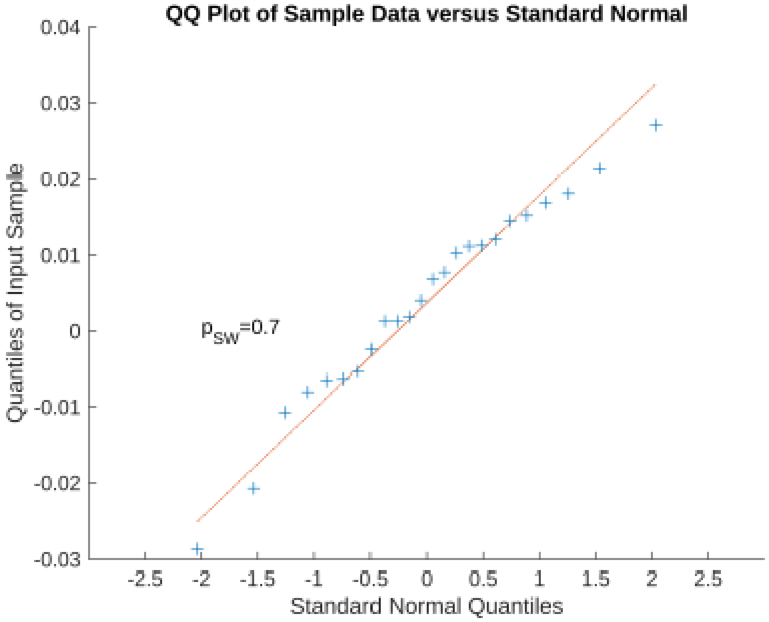

Spearman's Rho
ρ=0.28, p=0.09

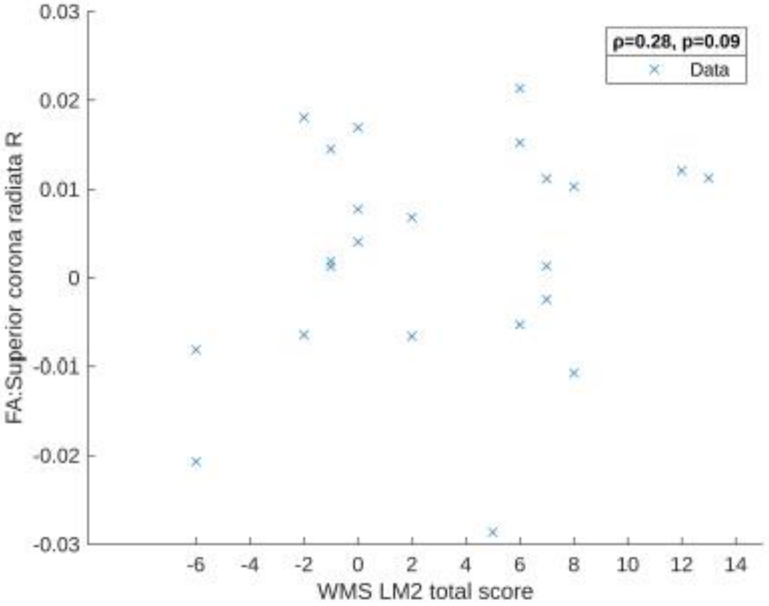

Posterior corona radiata R
FA~WMS LM2 total score

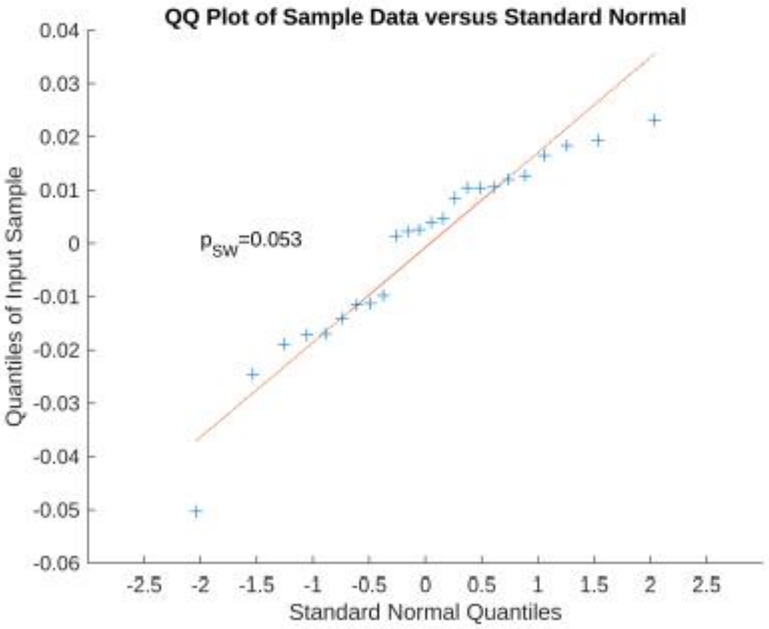

Spearman's Rho
 ρ=0.25, p=0.12
Posterior thalamic radiation R
FA~WMS LM2 total score

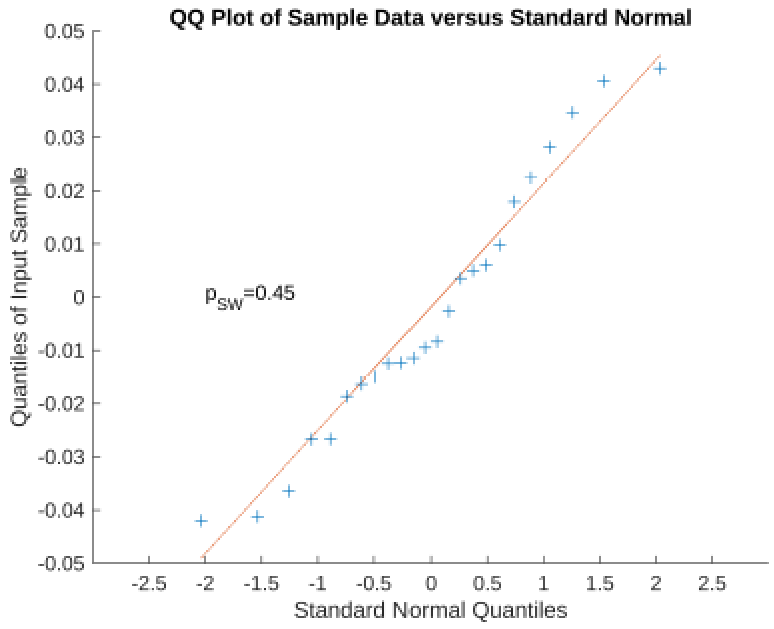

Spearman's Rho
ρ=0.3, p=0.077

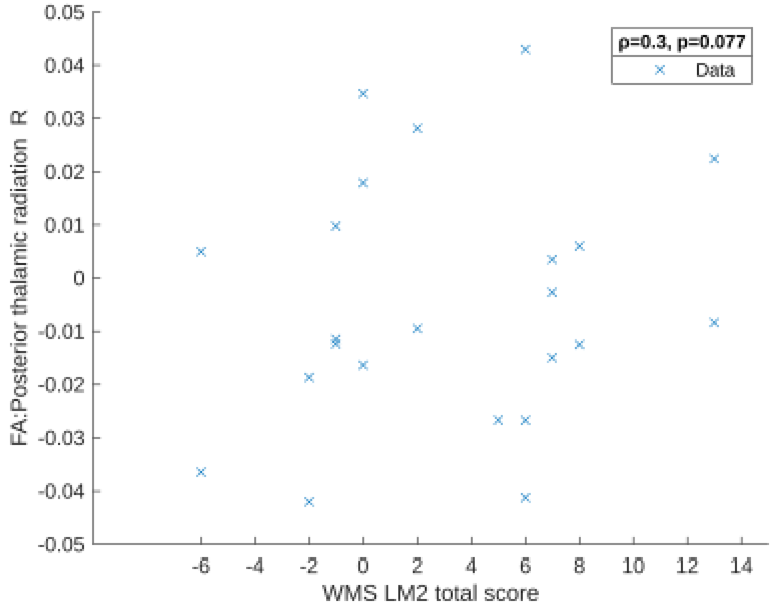

Sagittal stratum R
FA~WMS LM2 total score

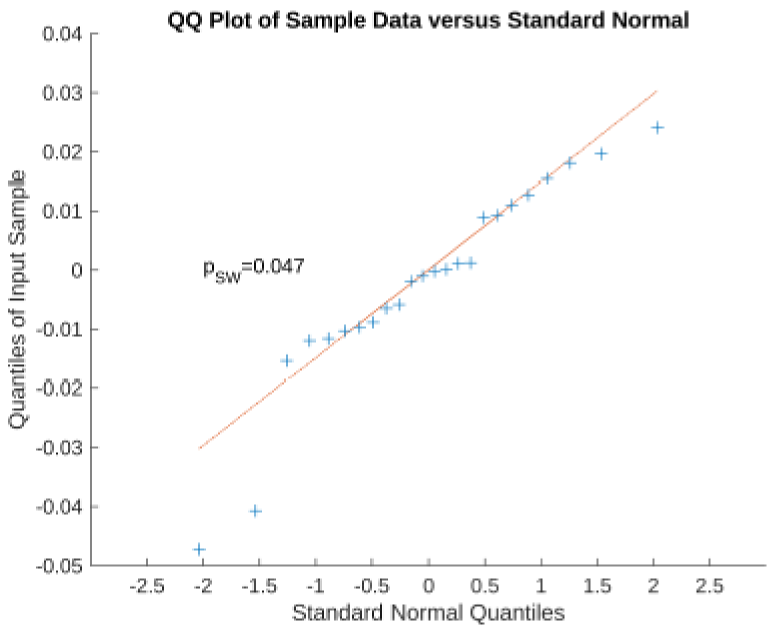

Spearman's Rho
ρ=0.31, p=0.07

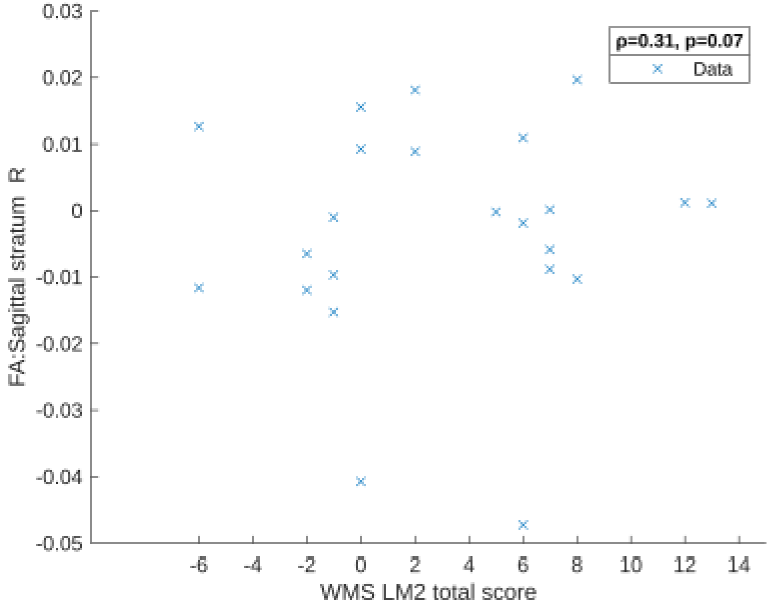

Superior longitudinal fasciculus R
FA~WMS LM2 total score

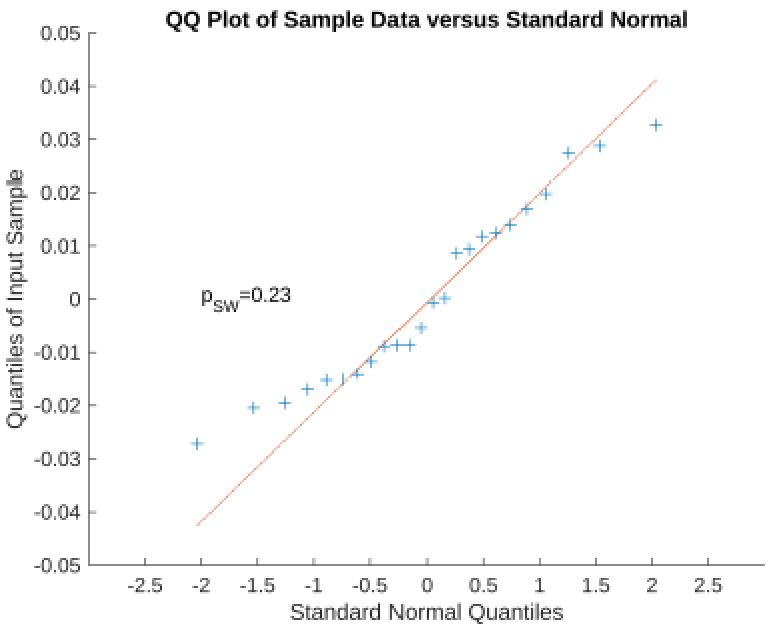

Spearman's Rho
ρ=0.38, p=0.034

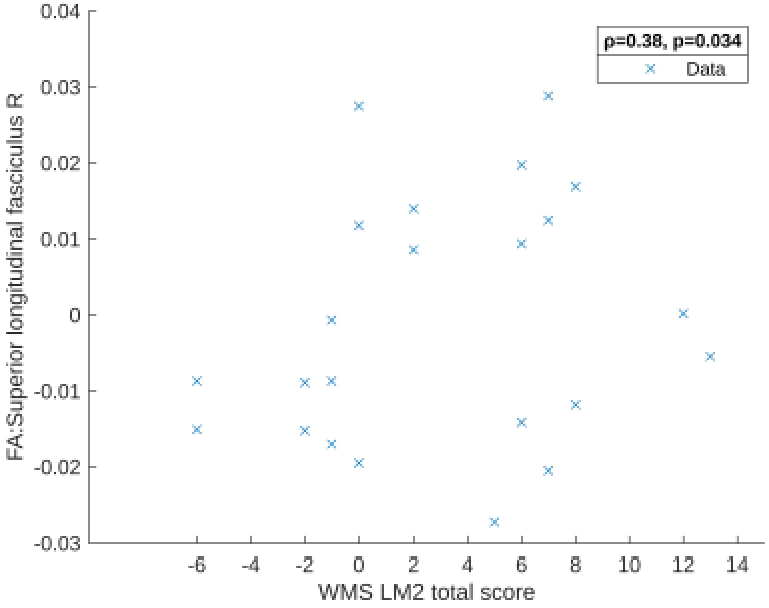

Tapetum R
FA~WMS LM2 total score

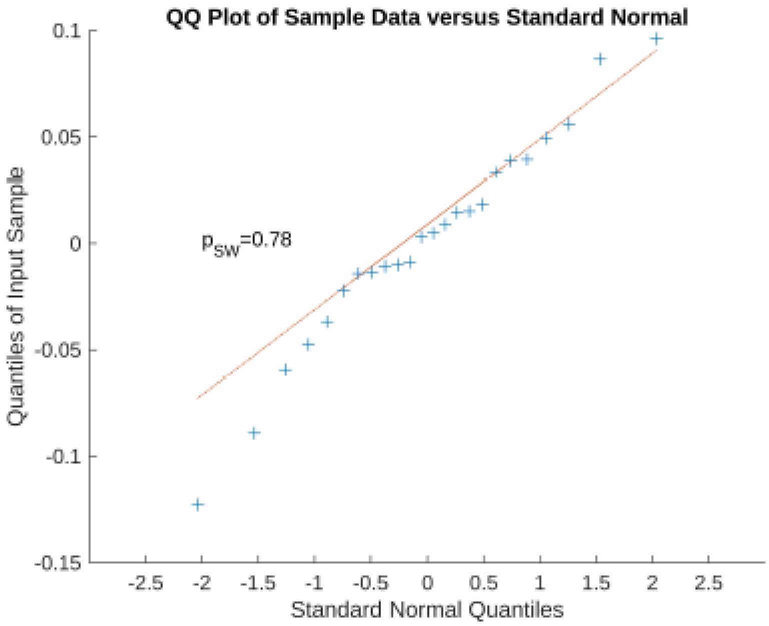

Spearman's Rho
ρ=0.42, p=0.02

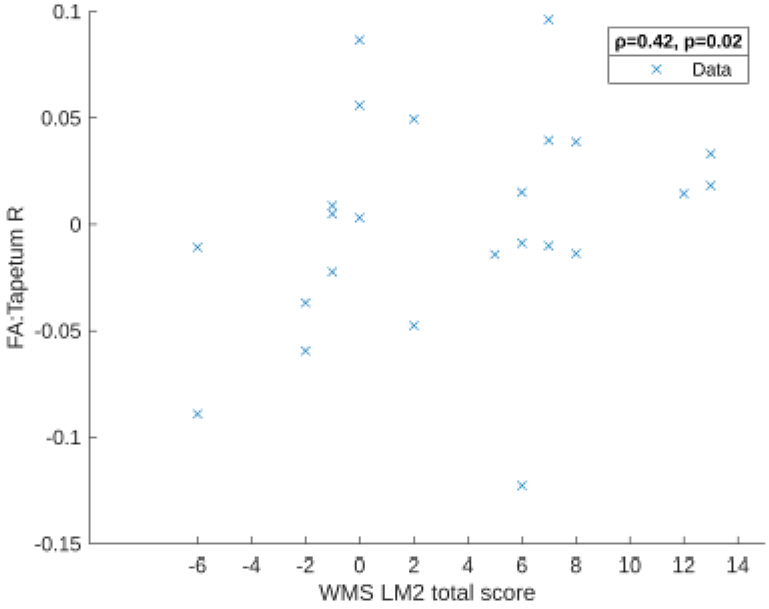

Body of corpus callosum
FA~Qmci score tot

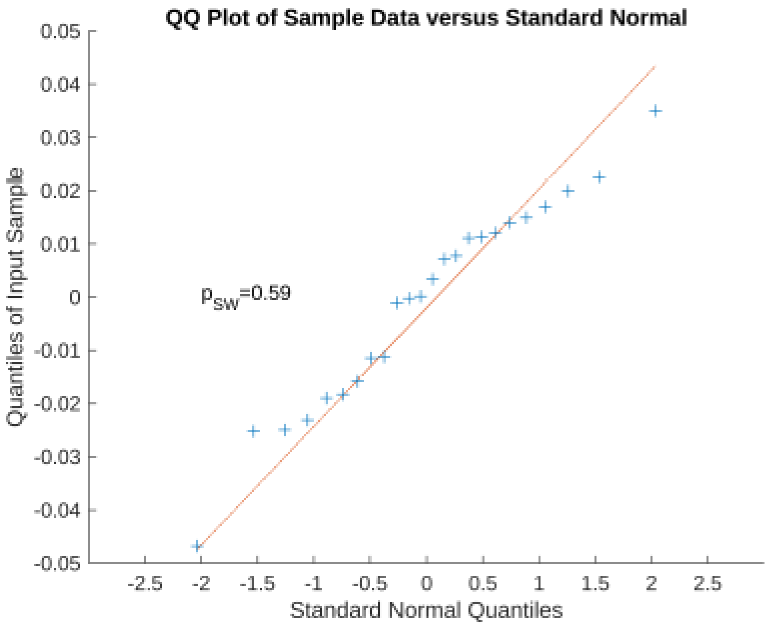

Pearson R
 r=0.27, p=0.099

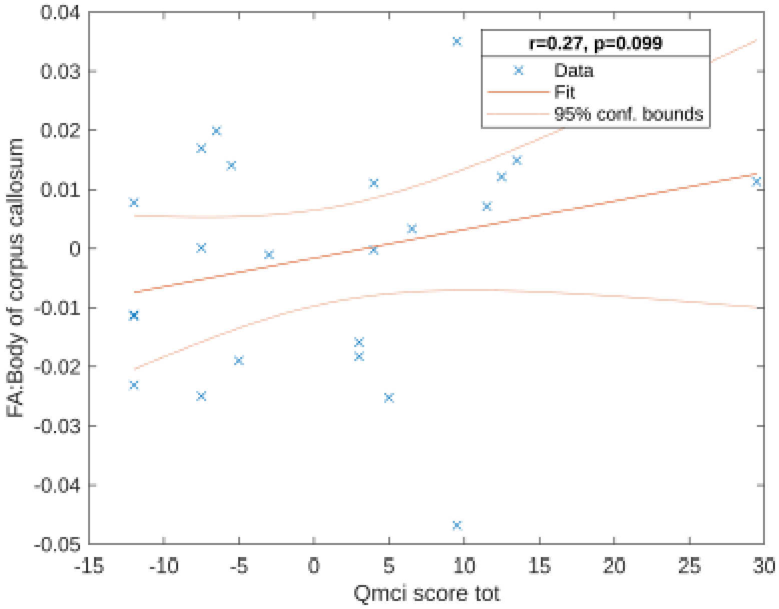

Splenium of corpus callosum
FA~Qmci score tot

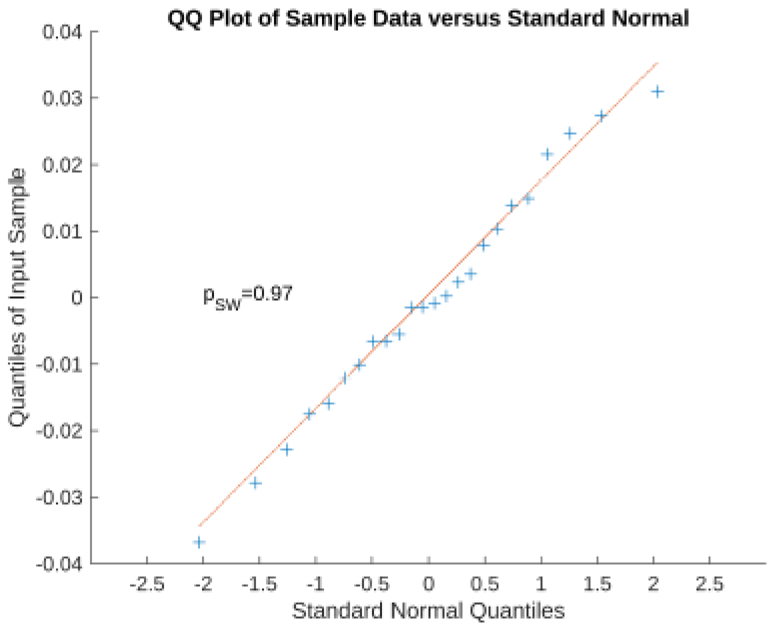


```
Pearson R
 r=0.39, p=0.03
```

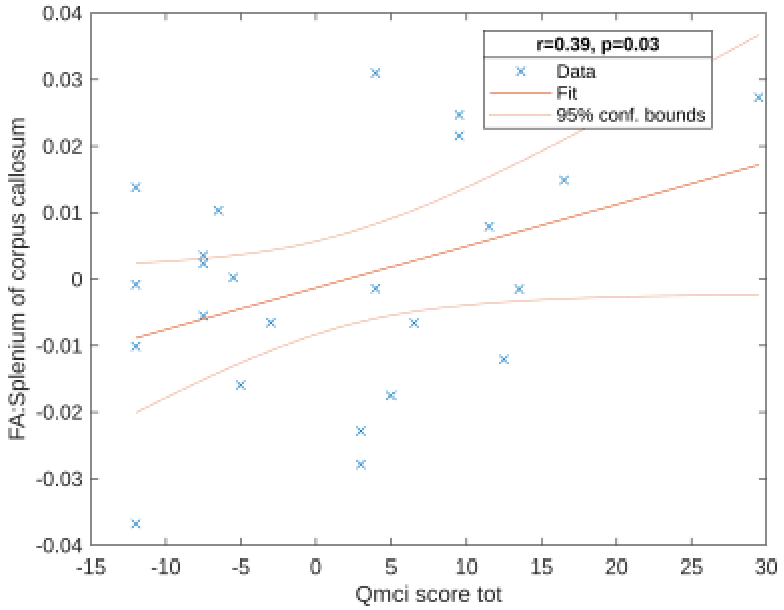


```
Retrolenticular part of internal capsule R
FA~Qmci score tot
```

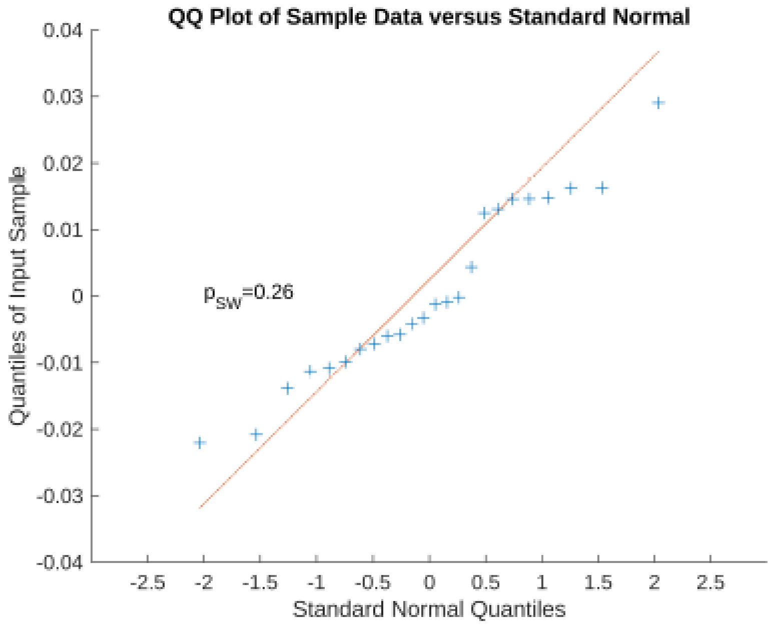


```
Pearson R
 r=-0.13, p=0.72
Superior corona radiata R
FA~Qmci score tot
```

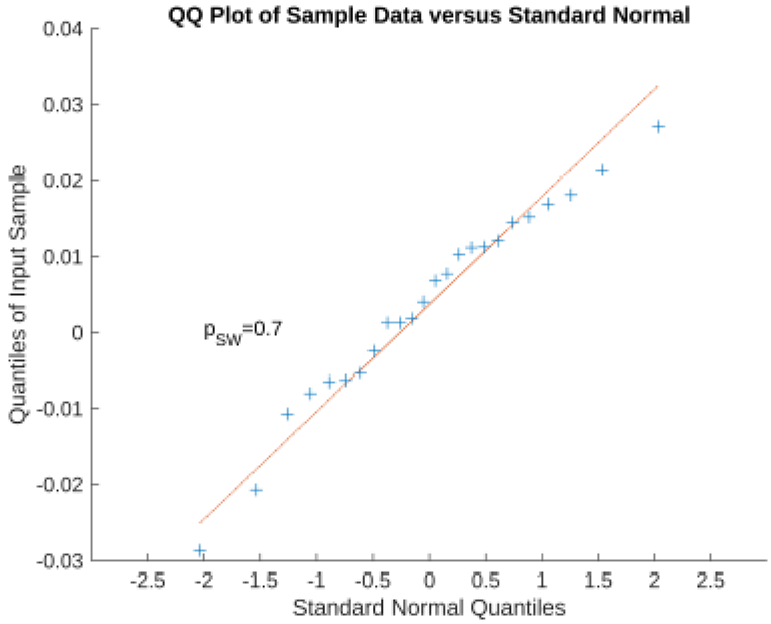


```
Pearson R
 r=0.089, p=0.34
Posterior corona radiata R
FA~Qmci score tot
```

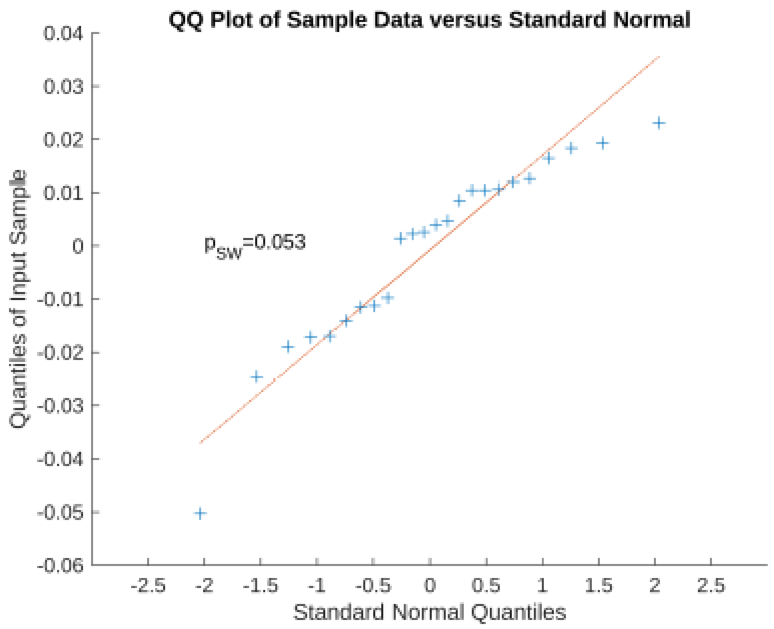

Pearson R
r=0.18, p=0.2
Posterior thalamic radiation R
FA~Qmci score tot

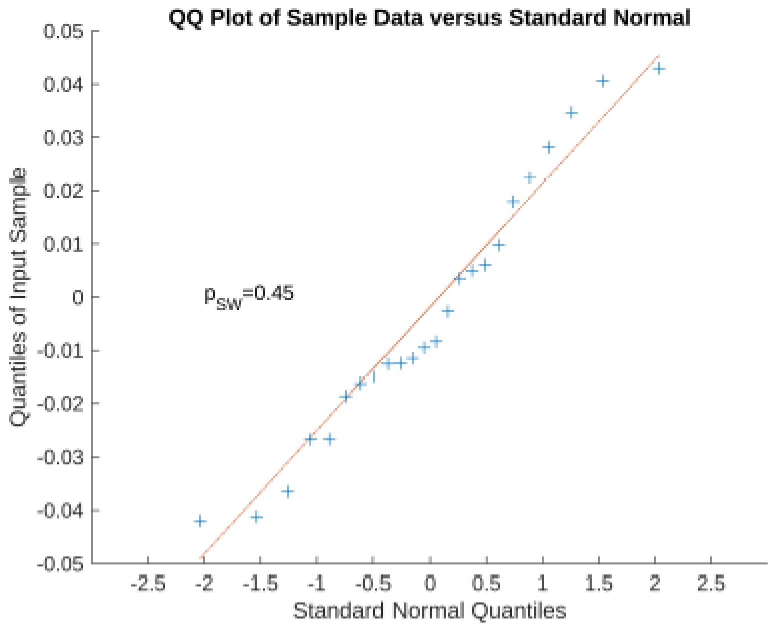

Pearson R
r=0.37, p=0.037

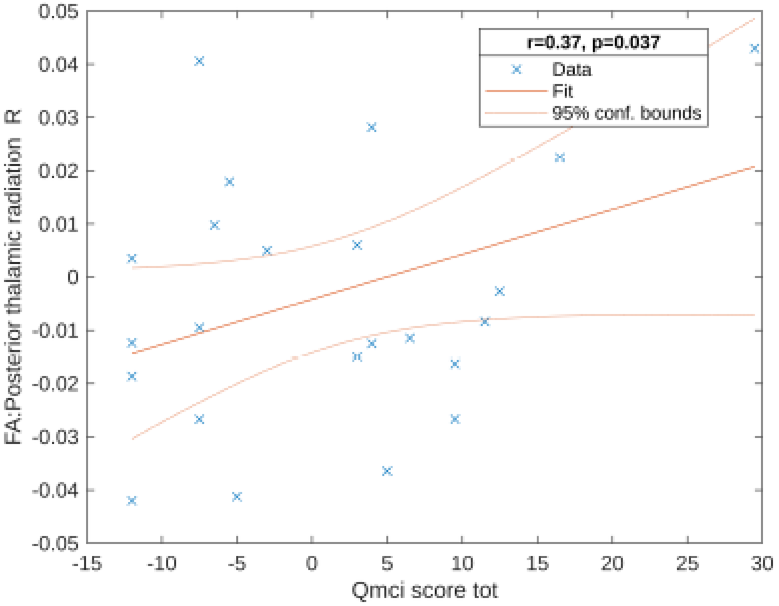


```
Sagittal stratum  R
FA~Qmci score tot
```

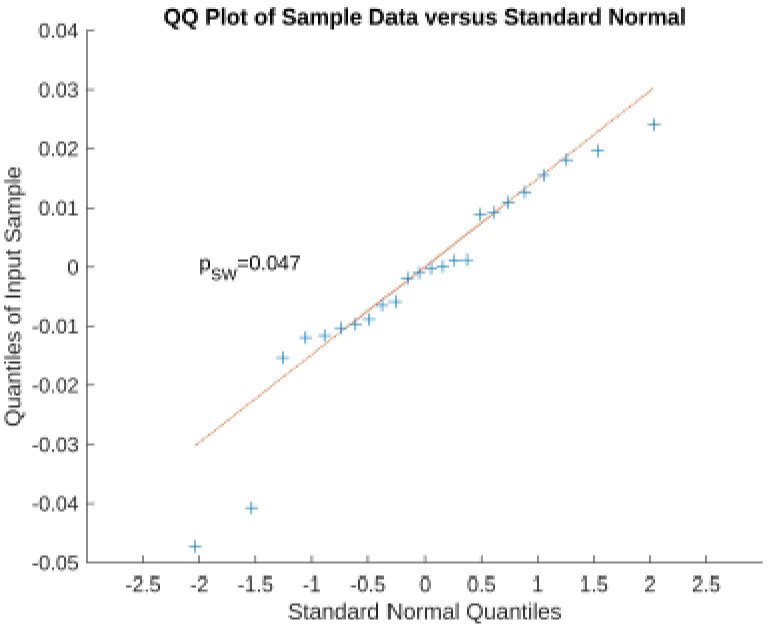


```
Spearman's Rho
 ρ=0.14, p=0.26
Superior longitudinal fasciculus R
FA~Qmci score tot
```

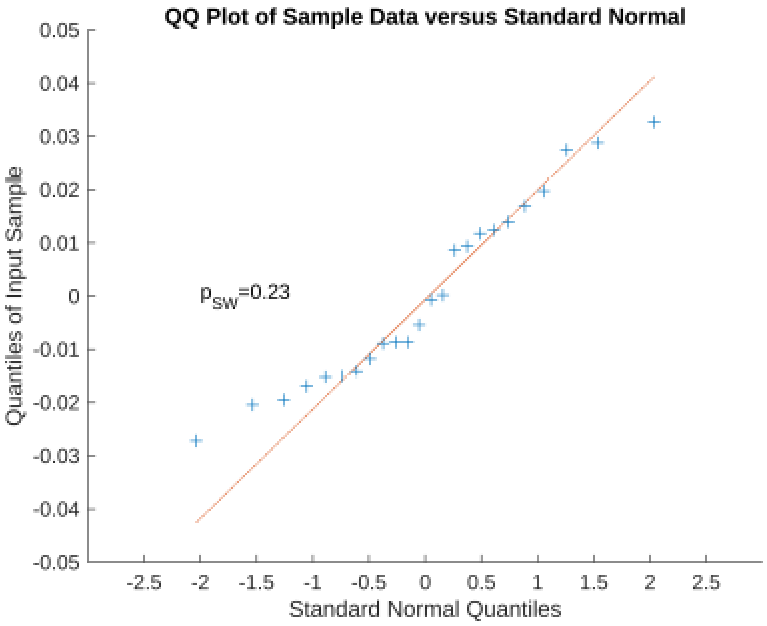

Pearson R
r=0.25, p=0.12
Tapetum R
FA~Qmci score tot

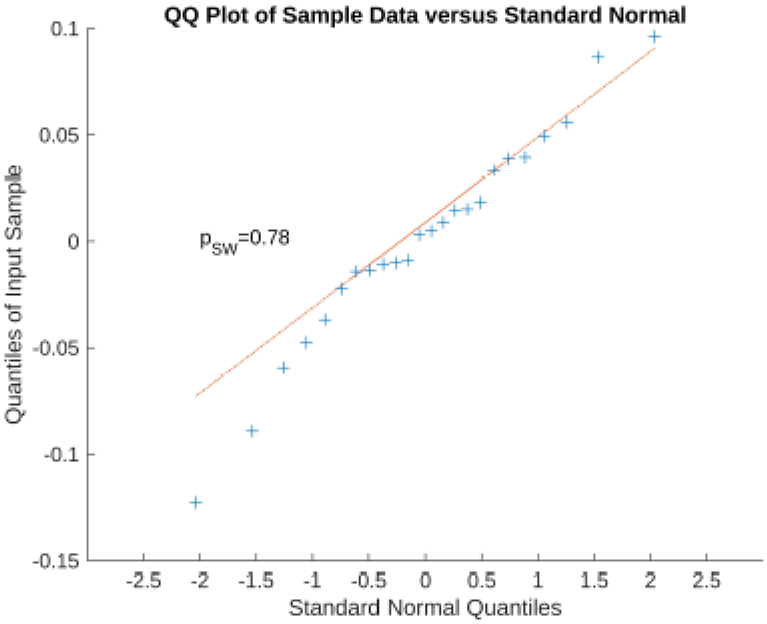

Pearson R
r=0.3, p=0.077

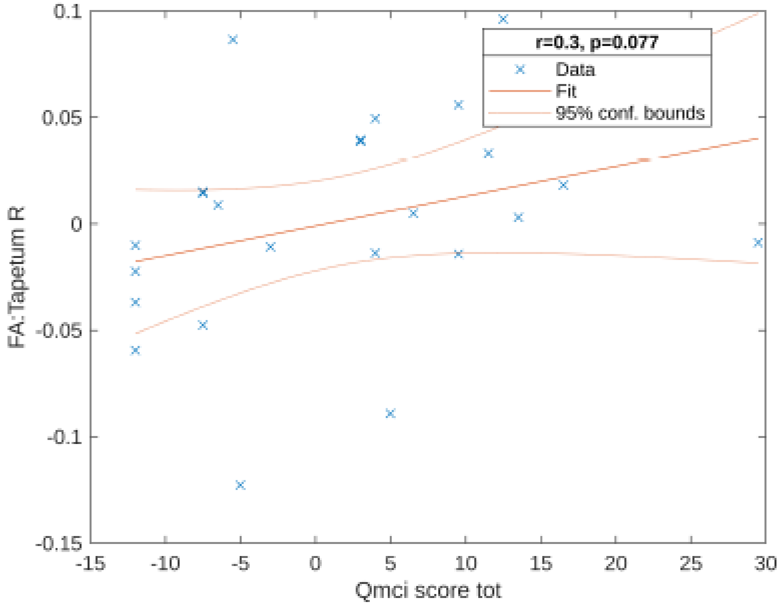


```
Body of corpus callosum
RD~WMS LM1 total score
```

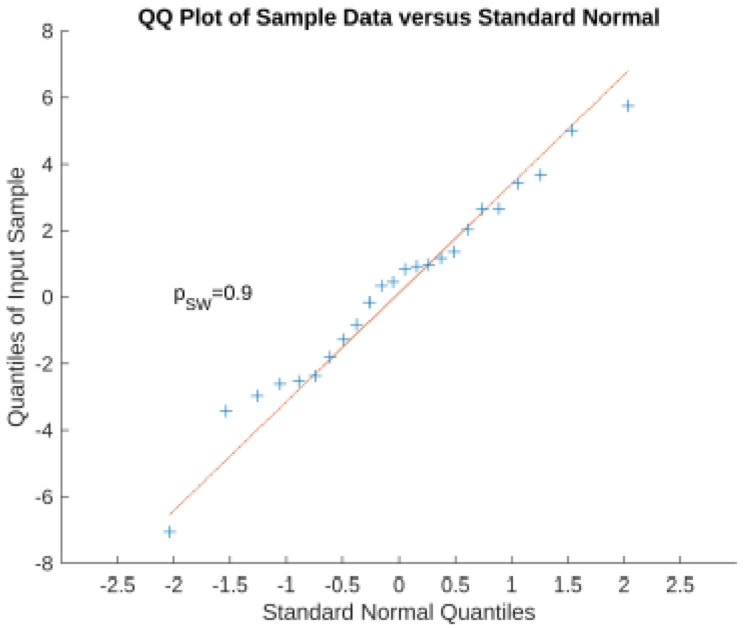


```
Pearson R
 r=0.11, p=0.7
Splenium of corpus callosum
RD~WMS LM1 total score
```

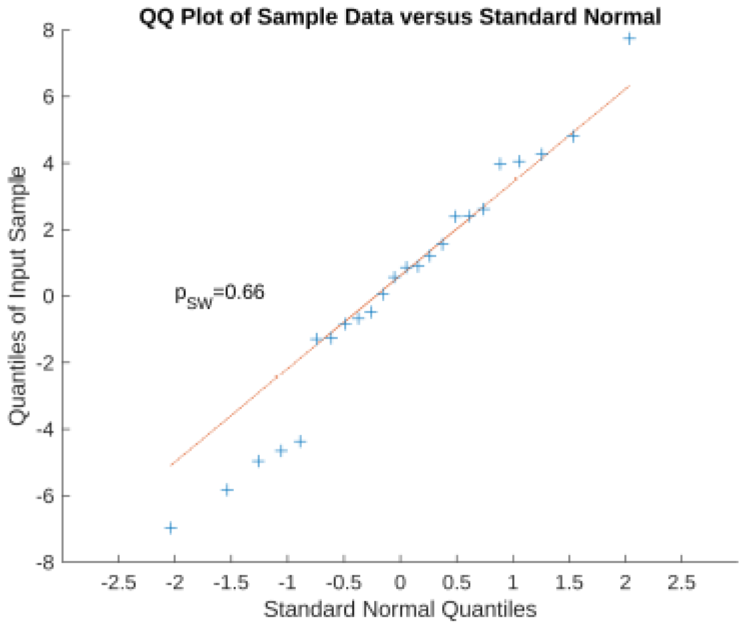

Pearson R
r=-0.038, p=0.43
Posterior corona radiata L
RD~WMS LM1 total score

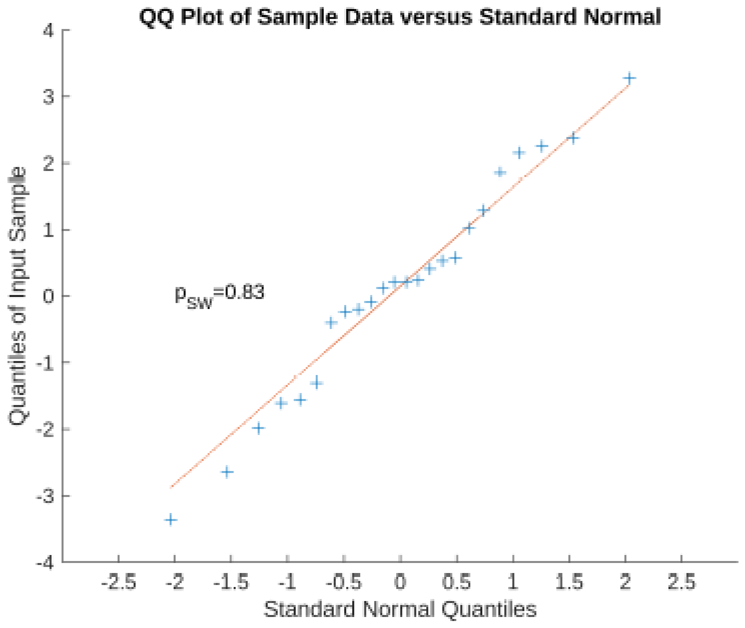

Pearson R
r=0.087, p=0.66
Posterior thalamic radiation L
RD~WMS LM1 total score

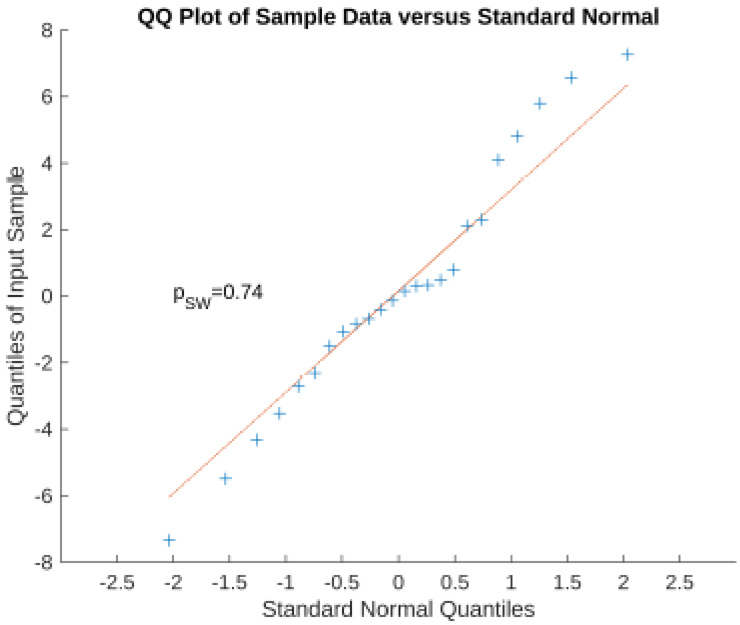

Pearson R
r=0.35, p=0.95
Tapetum L
RD~WMS LM1 total score

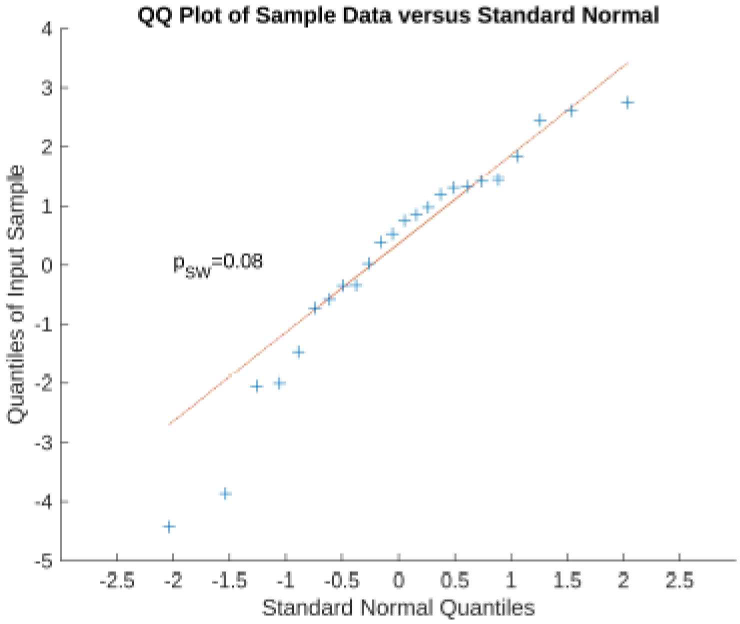

Pearson R
r=0.18, p=0.8
Body of corpus callosum
RD~WMS LM2 total score

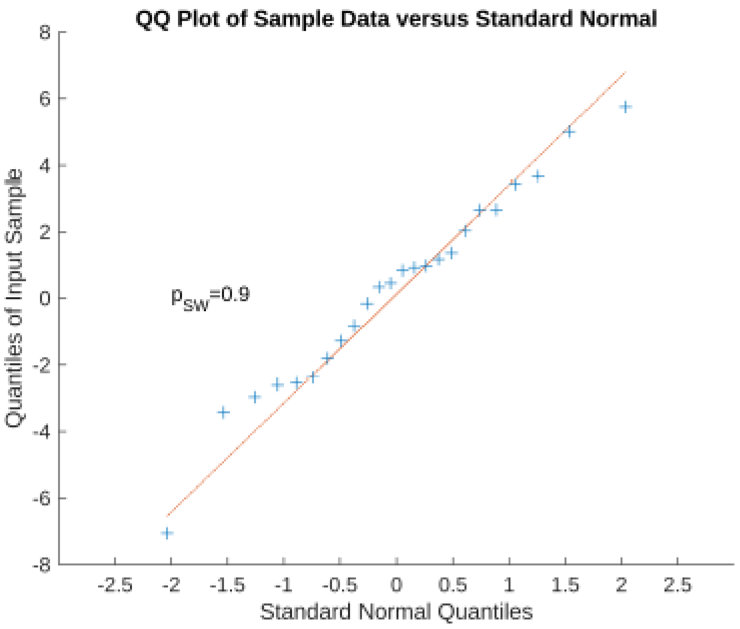

Spearman's Rho
ρ=-0.078, p=0.36
Splenium of corpus callosum
RD~WMS LM2 total score

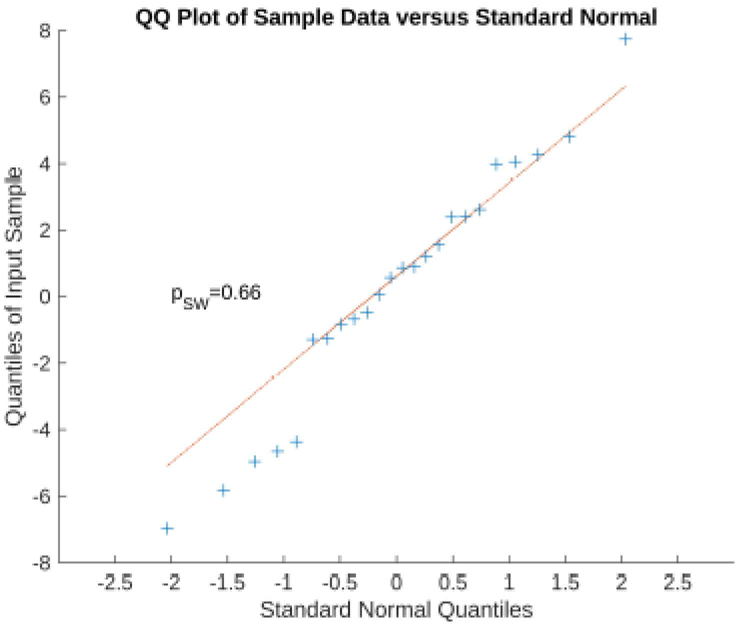

Spearman's Rho
ρ=-0.23, p=0.14
Posterior corona radiata L
RD~WMS LM2 total score

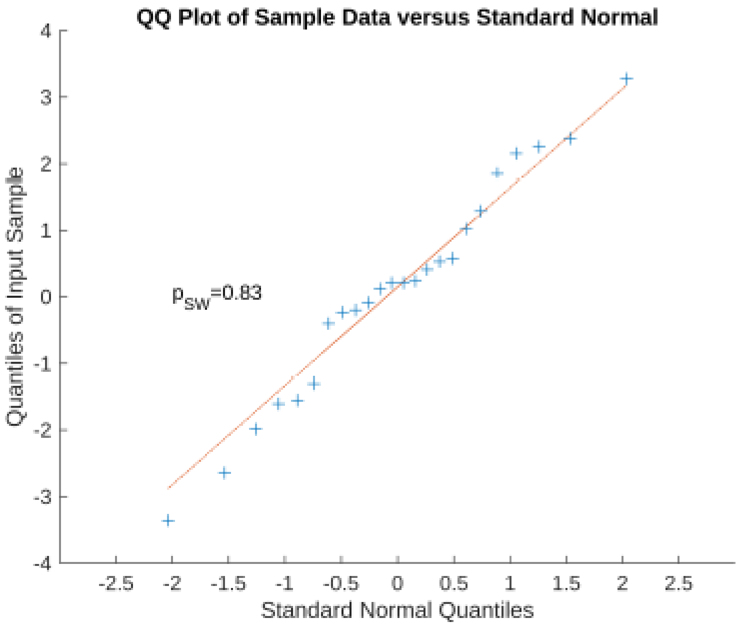

Spearman's Rho
ρ=-0.14, p=0.26
Posterior thalamic radiation L
RD~WMS LM2 total score

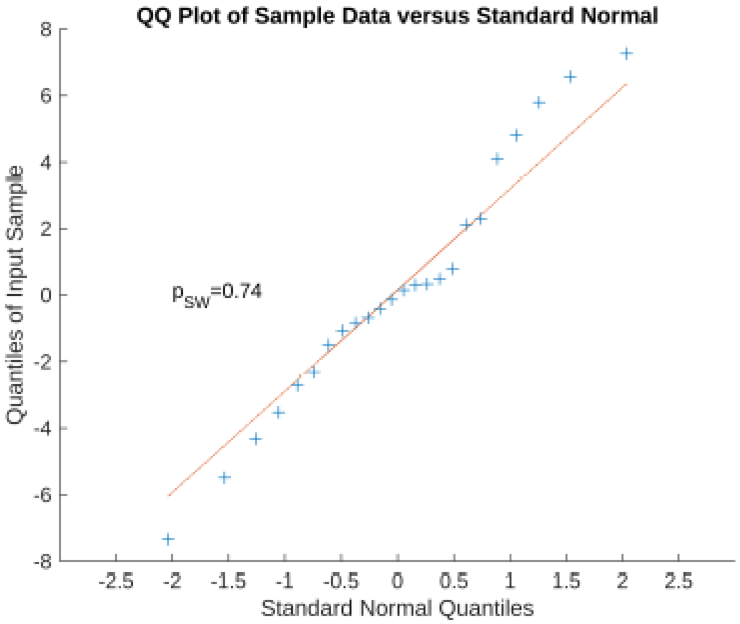

Spearman's Rho
ρ=-0.019, p=0.47
Tapetum L
RD~WMS LM2 total score

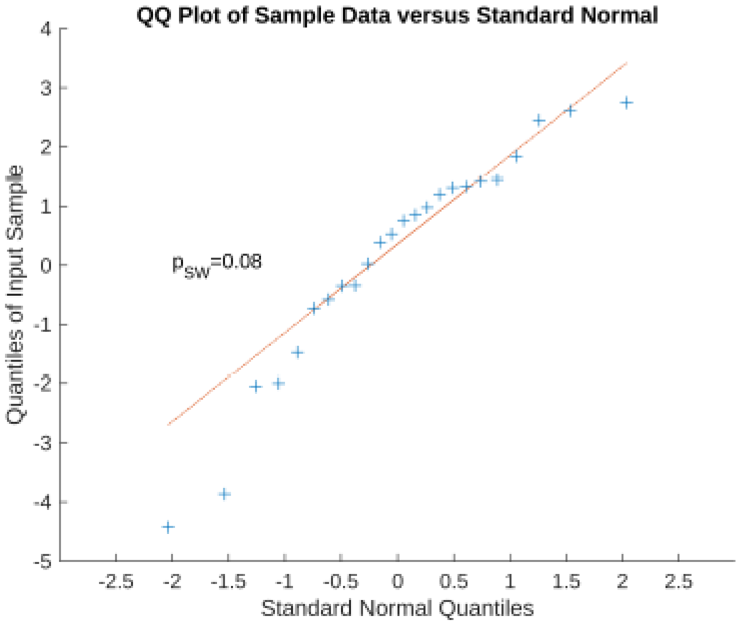

Spearman's Rho
ρ=-0.076, p=0.36
Body of corpus callosum
RD~Qmc1 score tot

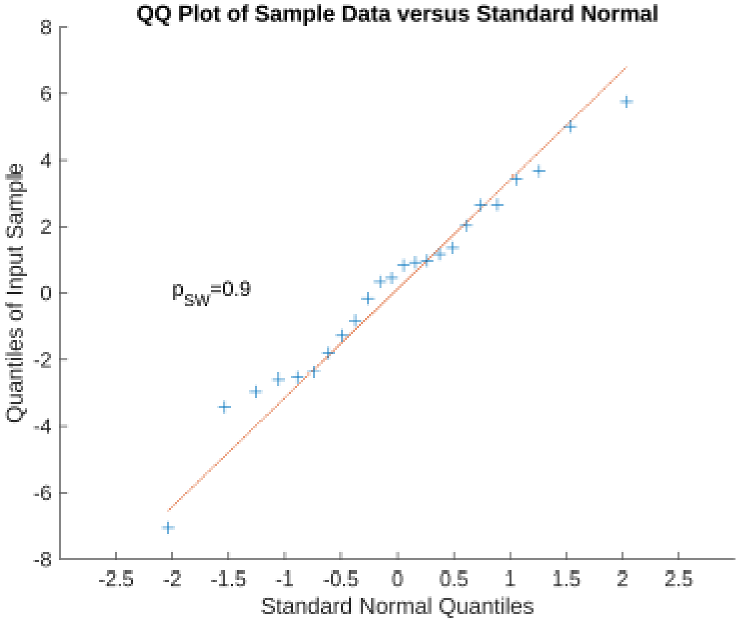

Pearson R
r=-0.38, p=0.034

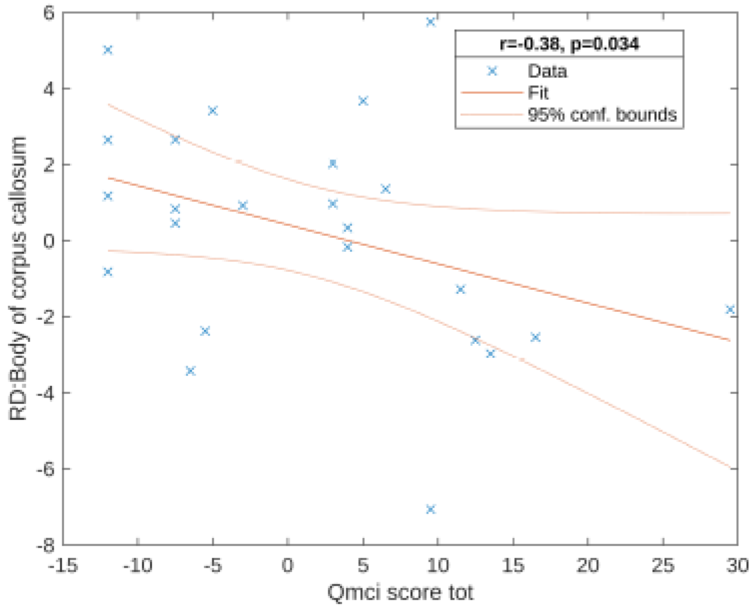


```
Splenium of corpus callosum
RD~Qmci score tot
```

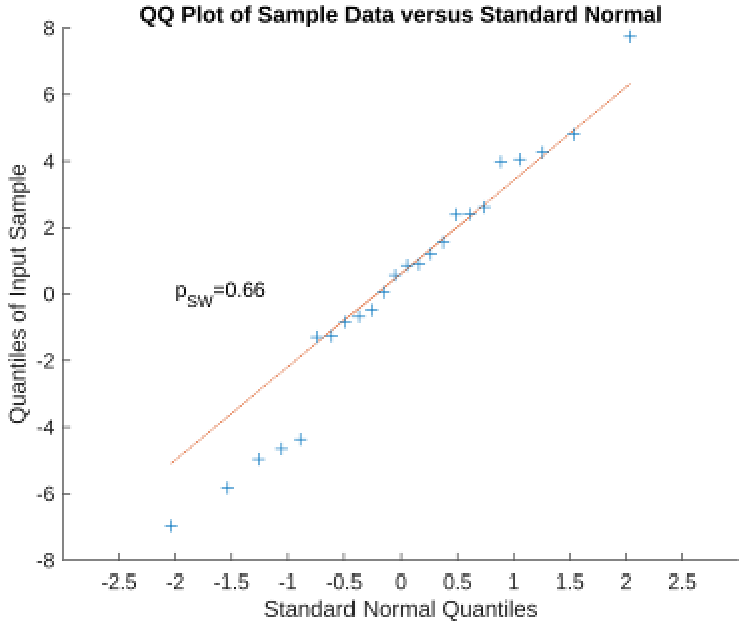


```
Pearson R
 r=-0.49, p=0.0074
```

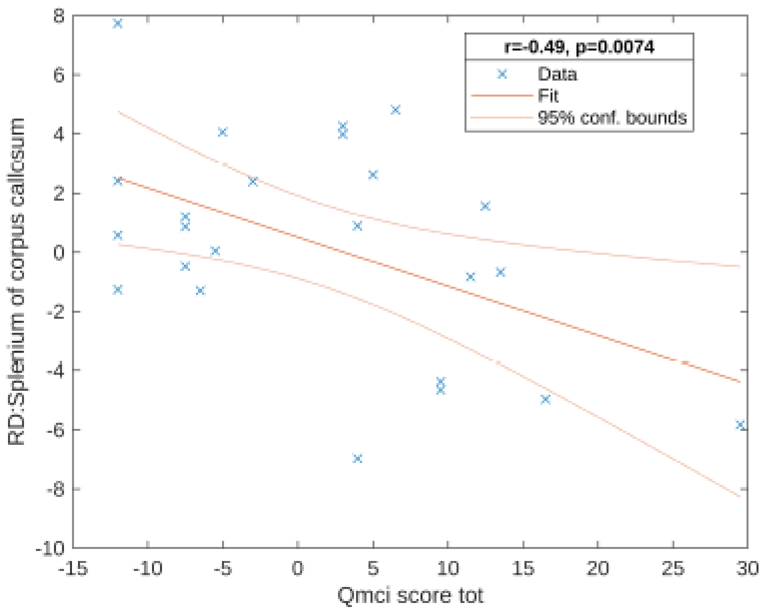


```
Posterior corona radiata L
RD~Qmci score tot
```

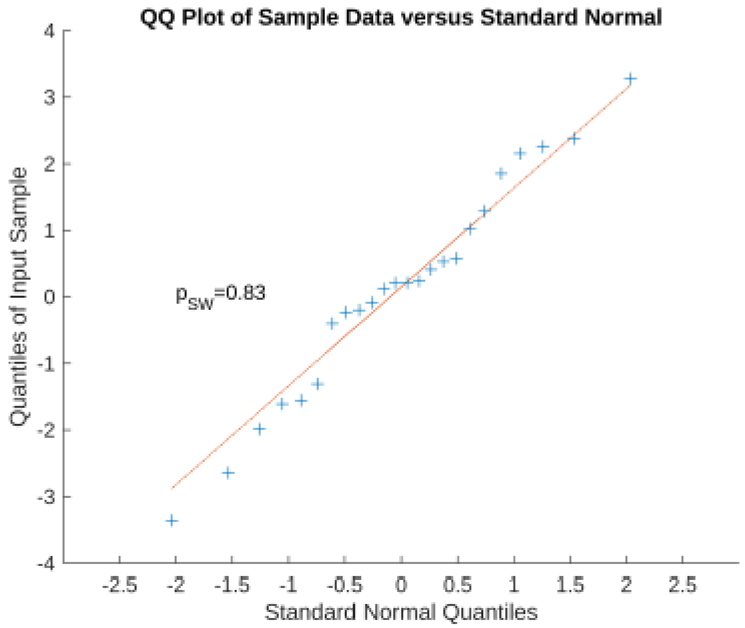


```
Pearson R
 r=-0.35, p=0.048
```

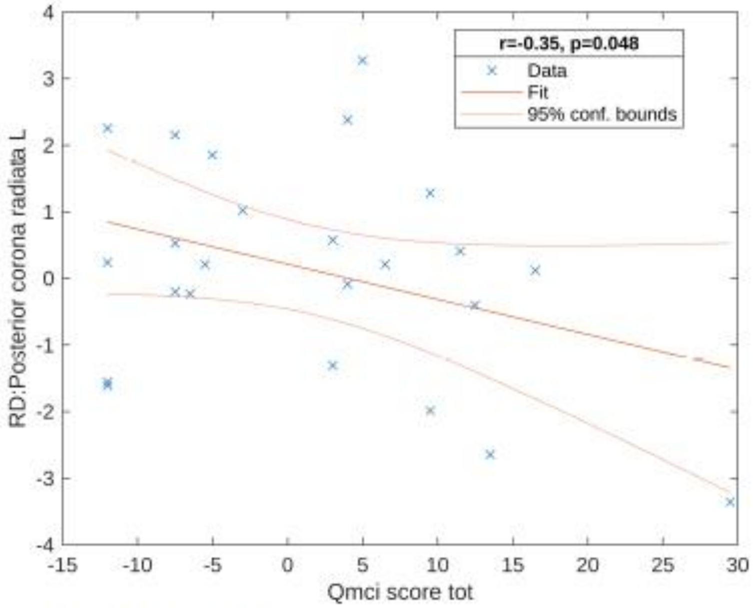

Posterior thalamic radiation L
RD~Qmci score tot

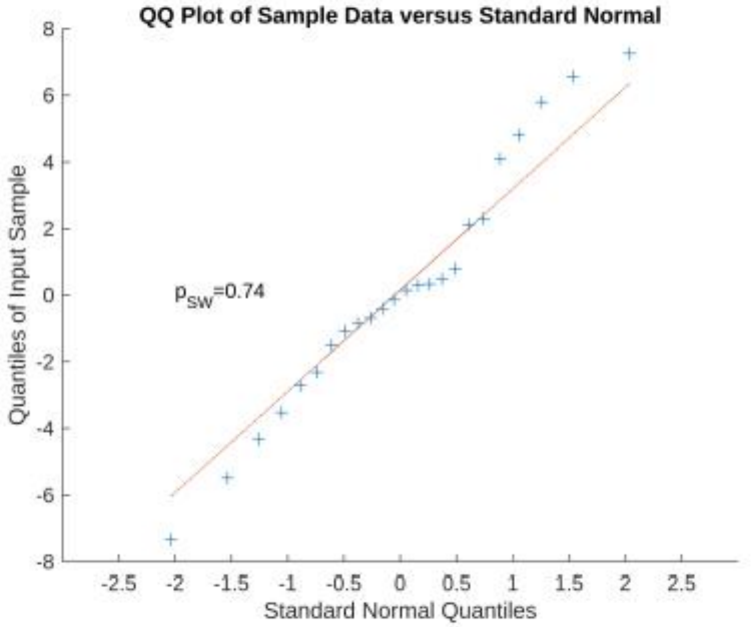

Pearson R
r=-0.15, p=0.25
Tapetum L
RD~Qmci score tot

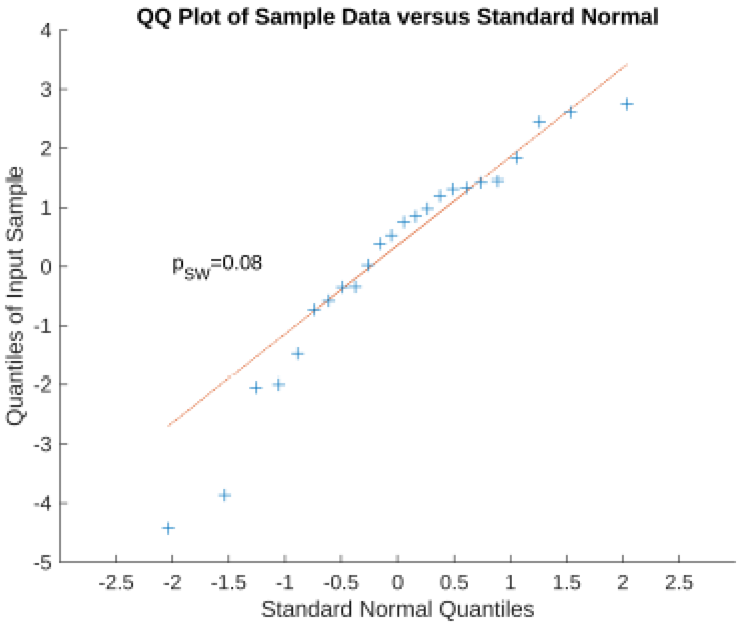


```
Pearson R
 r=-0.11, p=0.31
```

References

1. Boutron I, et al. CONSORT Statement for Randomized Trials of Nonpharmacologic Treatments: A 2017 Update and a CONSORT Extension for Nonpharmacologic Trial Abstracts. Annals of internal medicine. 2017;167(1):40-47. doi: https://doi.org/10.7326/M17-0046.
2. Manser P, et al. Effectiveness of an Individualized Exergame-Based Motor-Cognitive Training Concept Targeted to Improve Cognitive Functioning in Older Adults With Mild Neurocognitive Disorder: Study Protocol for a Randomized Controlled Trial. JMIR Resarch Protocols. 2023;12:e41173. doi: https://doi.org/10.2196/41173.
3. Manser P, de Bruin ED. “Brain-IT”: Exergame training with biofeedback breathing in neurocognitive disorders. Alzheimer's & dementia. 2024;20(7):4747-4764. doi: https://doi.org/10.1002/alz.13913.
